# Vespakinin-M delineates an AMPK/mTOR-arginine-TCA cycle axis to act as an immunometabolic switch in post-stroke microglia

**DOI:** 10.1016/j.redox.2026.104210

**Published:** 2026-05-12

**Authors:** Dexiao Wang, Jingyu Zhang, Zhejun Zhuang, Qian Wang, Jie Li, Xue Wang, Kunkun Li, Yunyun Liu, Yanhui Cao, Lijuan Li, Yunwu Zhang, Yu Zhao, Yingjun Zhao, Hairong Zhao, Chenggui Zhang

**Affiliations:** a Yunnan Provincial Key Laboratory of Entomological Biopharmaceutical R&D, College of Pharmacy, Dali University, Dali, Yunnan, 671000, China; b Department of Neurology and Department of Neuroscience, The First Affiliated Hospital of Xiamen University, Institute of Neuroscience, Fujian Provincial Key Laboratory of Neurodegenerative Disease and Aging Research, School of Medicine, Xiamen University, Xiamen, Fujian, 361005, China; c The First Affiliated Hospital of Dali University, Dali University, Dali, Yunnan, 671000, China; d National-Local Joint Engineering Research Center of Entomoceutics, Dali, 671000, China

**Keywords:** Vespakinin-M, Ischemic stroke, Microglia, Mitochondrial dysfunction, Immunometabolism, Arginine metabolism

## Abstract

Despite advances in recanalization therapy for ischemic stroke, effective neuroprotection against cerebral ischemia-reperfusion injury (CIRI) remains an unmet need, largely due to persistent microglia-driven neuroinflammation and associated oxidative stress. Vespakinin-M (VK) is a naturally neuroprotective peptide isolated from wasp venom that can cross the blood-brain barrier. Although VK has been shown to improve functional outcomes in preliminary stroke models, its underlying mechanisms remain unclear. Here, we show that administration of VK alleviates neuroinflammation and oxidative damage in a mouse stroke model. This neuroprotection is orchestrated by microglial metabolic reprogramming, which shifts their energy metabolism from aerobic glycolysis toward oxidative phosphorylation (OXPHOS) and their functional phenotype from pro-inflammatory M1 to reparative M2. Integrated multi-omics and isotopic tracing uncover that VK redirects arginine metabolism to generate fumarate. This directly couples amino acid catabolism with the tricarboxylic acid (TCA) cycle, thereby restoring mitochondrial bioenergetics and redox balance. Mechanistically, VK activates the energy sensor AMPK while inhibiting the anabolic regulator mTOR. AMPK knockdown partially abolishes the beneficial effects of VK, establishing the AMPK/mTOR axis as the upstream regulator of this arginine-centric metabolic rewiring. Interestingly, VK retains the ability to stimulate *de novo* arginine synthesis even under arginine-deprived conditions, and its efficacy is synergistically enhanced with arginine supplementation. Together, these findings define an immunometabolic axis—AMPK/mTOR-arginine-TCA cycle coupling—that dictates microglial fate after stroke, and suggests VK as a therapeutic agent capable of concurrently targeting neuroinflammation, mitochondrial dysfunction, and metabolic imbalance.

## Highlights

• Vespakinin-M shifts post-stroke microglia from glycolysis to OXPHOS.
• It promotes M1-to-M2 polarization and reduces neuroinflammation.
• Mechanistically, it restores the arginine-TCA cycle via AMPK/mTOR.

## Graphical abstract

**Figure:**
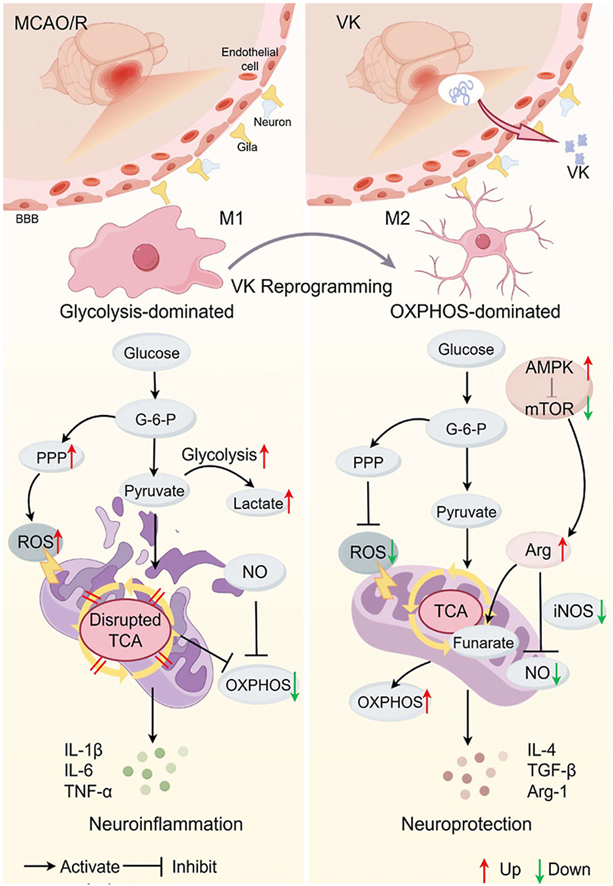

## 1 Introduction

Ischemic stroke is a leading cause of death and long-term disability worldwide [1]. Even when blood flow is restored, patients often suffer from cerebral ischemia-reperfusion injury (CIRI). Beyond the initial ischemic insult, persistent neuroinflammation driven by overactivated microglia—the brain's resident immune cells—contributes to progressive tissue damage [2] and lasting neurological deficits [3]. Microglia play a dual role: classical M1 polarization exacerbates neuronal injury by releasing pro-inflammatory cytokines and reactive oxygen species (ROS), whereas alternative M2 polarization supports tissue repair and resolves inflammation [4,5]. Current recanalization therapies, such as thrombolysis, fail to address this maladaptive immune response [6,7], underscoring an urgent need for strategies that precisely modulate microglial phenotype.

Recent advances in immunometabolism have established that microglial function is closely linked to their metabolic state [8,9]. Pro-inflammatory M1 microglia undergo a metabolic shift toward aerobic glycolysis (analogous to the Warburg effect), which supports rapid ATP generation and biosynthetic precursor supply but also leads to lactate accumulation [10], ROS overproduction [11], and a toxic microenvironment [12]. Conversely, anti-inflammatory M2 microglia primarily rely on oxidative phosphorylation (OXPHOS) and fatty acid oxidation, generating sustained energy and supporting reparative functions [13]. Thus, metabolic reprogramming directly determines microglial fate and redox balance.

Notably, amino acid metabolism—particularly that of arginine—serves as a critical regulator at this intersection. Arginine metabolism bifurcates into distinct pathways: nitric oxide synthase (NOS)-mediated production of NO versus arginase-1 (Arg1)-mediated production of ornithine and polyamines [14]. Moreover, arginine metabolism can directly feed into the tricarboxylic acid (TCA) cycle through the argininosuccinate lyase (ASL)-catalyzed reaction, which generates fumarate as a key intermediate. This metabolic node establishes a direct functional link between amino acid catabolism and mitochondrial OXPHOS [15]. The AMPK/mTOR signaling axis integrates energy status and nutrient sensing to govern this immunometabolic landscape. AMPK activation under energy stress inhibits mTOR, a key promoter of anabolism [16,17]. In microglia, this balance dictates metabolic preference and functional polarization: AMPK activation with mTOR inhibition favors oxidative metabolism and an M2 phenotype, whereas the opposite pattern supports glycolytic metabolism and M1 polarization [18].

Despite remarkable advances in delineating the immunometabolic basis of ischemic stroke, fundamental gaps persist in our ability to translate these insights into effective therapies. First, few pharmacological agents have been shown to actively reverse the pathological metabolic rewiring that drives sustained neuroinflammation and neuronal damage after stroke. Second, the cell-intrinsic role of arginine metabolism in regulating post-ischemic microglial polarization and its therapeutic potential to restore mitochondrial energy production are not fully defined. Third, the AMPK/mTOR axis is established as an upstream regulator of the glycolysis–OXPHOS balance, but its function in coupling therapeutic intervention to arginine metabolism and downstream TCA cycle after stroke has not been directly demonstrated.

Vespakinin-M (VK), a naturally bioactive peptide isolated from the venom of *Vespa magnifica* Smith, has confirmed efficacy in preliminary stroke models, as evidenced by reducing infarct volume, preserving the blood-brain barrier (BBB) integrity, and improving functional outcomes [19]. Here, using integrated multi-omics, ^13^C-stable isotope tracing, and genetic approaches, we describes that VK orchestrates microglial immunometabolism via regulating the AMPK/mTOR pathway, thereby reconstructing the arginine-TCA cycle axis to restore redox balance and promote an anti-inflammatory phenotype.

## 2 Materials and methods

### 2.1 Animals and ethical approval

This study used adult male C57BL/6 mice (weighing 20−25 g and aged 8−10 weeks) obtained from Hunan Silaike Jingda Laboratory Animal Co., Ltd. (License No.: SYXK (Dian) 2024-0001). Mice were housed in the Experimental Animal Center of Dali University under controlled conditions: temperature maintained at 22 °C ± 2 °C, a 12-h light/dark cycle, and free access to food and water. All experimental procedures were approved by the Animal Welfare and Ethics Committee of Dali University, China (Approval No.: 2022-PZ-40) and conducted in accordance with institutional and national guidelines. A total of 395 mice were used, with detailed group allocation provided in Supplementary Table 1.

### 2.2 Establishment of the MCAO model and exclusion criteria

Focal CIRI was constructed by middle cerebral artery occlusion (MCAO), as described previously with minor modifications [20,21]. Briefly, mice were anesthetized with 1.5% isoflurane (#R510-22-10; RWD Life Science, China) delivered in a gas mixture of 70% nitrous oxide (N_2_O) and 30% oxygen (O_2_). A silicone-coated 6-0 nylon monofilament suture (diameter: 0.21 ± 0.02 mm; #MSMC21B120PK50; RWD Life Science, China) was inserted into the external carotid artery and advanced through the internal carotid artery to occlude the origin of the middle cerebral artery for 60 min. The filament was then withdrawn for reperfusion (MCAO/R). Sham-operated mice underwent identical surgical procedures except for filament insertion. Body temperature was maintained at 36.5 °C ± 0.5 °C using a feedback-controlled heating pad.

Mice were excluded [22,23] based on either a low or a maximally severe neurological score (<1 or = 4) at 3 h post-reperfusion, or the presence of subarachnoid hemorrhage at necropsy.

### 2.3 Experimental groups and drug treatment

Mice were randomly assigned to three groups: sham group (surgical procedure without MCAO), vehicle group (subjected to MCAO/R and administered an equivalent volume of phosphate-buffered saline [PBS]), and VK group (subjected to MCAO/R and treated with VK). After successful MCAO/R (neurological score 1−3), mice in the VK group received an intraperitoneal (*i.p.*) loading dose of VK (150 μg/kg in PBS) within 1 h post-reperfusion, followed by daily *i.p.* injections of VK (150 μg/kg/day) for 7 consecutive days. Vehicle and sham groups received isovolumetric PBS on the same schedule. The dosage was selected based on our previous study [19].

### 2.4 Tissue and sample collection

At designated time points (days 1, 3, 7, 11, and 14 post-MCAO/R), mice were deeply anesthetized using 3% isoflurane delivered via a precision vaporizer in a mixture of 70% N_2_O and 30% O_2_. Serum was collected via retro-orbital bleeding and stored at −80 °C. For biochemical assays, brains were rapidly removed without perfusion. The ischemic hemisphere was dissected on ice, snap-frozen, and stored at −80 °C. For histology, mice were transcardially perfused with PBS followed by 4% paraformaldehyde (PFA). Brains were post-fixed in PFA, cryoprotected, and sectioned. All subsequent analyses were performed with investigators blinded to group allocation.

### 2.5 Immunofluorescence staining

Brain tissue sections were processed as previously described [24]. Specifically, coronal brain sections (24 μm) were equilibrated to room temperature (RT), baked at 60 °C for 3 h, and fixed in 4% PFA for 20 min at RT. Antigen retrieval was performed in preheated sodium citrate buffer (10 mM, pH 6.0; #MVS-0101, Maxim Biotechnologies, China) at 95 °C for 20 min. After cooling, sections were permeabilized and blocked for 1 h at RT in a solution containing 0.3 M glycine, 0.1% Triton X-100, 10% donkey serum (#G1217, Servicebio, China), and 5% bovine serum albumin (BSA; #FC0077, MP Biomedicals, USA).

Sections were then incubated overnight at 4 °C with the following primary antibodies diluted in blocking solution: goat anti-ionized calcium-binding adapter molecule 1 (IBA1; 1:600, #ab5076, Abcam, UK), mouse anti-iNOS (1:200, #sc-7271, Santa Cruz Biotechnology, USA), rabbit monoclonal anti-Arg1 (1:200, #93668, Cell Signaling Technology [CST], USA), mouse anti-NeuN (1:800, #MAB377, Sigma-Aldrich, USA), rabbit anti-glucose transporter 1 (GLUT1; 1:100, #ab652, Abcam, UK), and rabbit anti-phosphofructokinase-2/fructose-2,6-bisphosphatase 3 (PFKFB3; 1:100, #13763-1-AP, Proteintech, USA). After washing, sections were incubated for 1 h at RT with species-specific secondary antibodies conjugated to Alexa Fluor® dyes (1:500; Abcam, UK): Donkey anti-rabbit immunoglobulin G (IgG) (AF405/ab175651, AF488/ab150073, AF594/ab150064), Donkey anti-mouse IgG (AF555/ab150110), Donkey anti-rabbit IgG (AF647/ab150075), and Donkey anti-goat IgG (AF488/ab150129). Nuclei were counterstained with 4′,6-diamidino-2-phenylindole (5 μg/mL; #D9542, Sigma-Aldrich, USA) for 5 min, and mounted with antifade medium (#F4680, Sigma-Aldrich, USA). Whole-slide imaging was performed using an Olympus VS200 slide scanner under identical exposure settings. Multispectral images were captured using identical exposure settings across all samples. Image processing (background subtraction, channel alignment) was conducted using Olympus OlyVIA v3.0 software.

### 2.6 Immunohistochemical staining

For immunohistochemistry, sections were prepared and subjected to antigen retrieval using the same protocol as described for immunofluorescence (Section 2.5). Endogenous peroxidase activity was then blocked by incubation with 3% hydrogen peroxide in methanol for 20 min at RT. After permeabilization with 1% Triton X-100 and blocking, sections were incubated overnight at 4 °C with rabbit anti-IBA1 primary antibody (1:400, #019-19741, FUJIFILM Wako Pure Chemical Corporation, Japan).

After washing, sections were incubated with biotinylated goat anti-rabbit IgG (1:100; #A0277, Beyotime Biotechnology, China) at 37 °C for 1 h, then with HRP-conjugated streptavidin (1:100; #A0303, Beyotime Biotechnology, China) at 37 °C for 40 min. Following 3,3′-diaminobenzidine development, sections were counterstained with hematoxylin. After dehydration and clearing, sections were mounted for analysis. IBA1-positive immunoreactivity was quantified in a blinded manner using ImageJ (NIH) on ≥ 3 fields per section with threshold-based segmentation.

### 2.7 Immunoblotting analysis

Protein lysates were prepared from the ischemic hemispheres or cultured cells using RIPA lysis buffer (50 mM Tris-HCl, pH 7.4; 150 mM NaCl; 1% NP-40; 0.5% sodium deoxycholate; and 0.1% SDS) supplemented with 1 mM phenylmethylsulfonyl fluoride and a protease inhibitor cocktail (#K0010, #K0022, MedChemExpress, USA). Lysates were centrifuged at 12,000×*g* for 15 min at 4 °C. The protein concentration of the supernatant was determined using a bicinchoninic acid (BCA) protein assay kit (#B5001, LABLEAD, China), with BSA as the standard. Protein samples (40 μg/lane) were separated on 8%−12% SDS-polyacrylamide gels alongside prestained molecular weight markers (#P1018, LABLEAD, China), and subsequently transferred to polyvinylidene difluoride membranes (0.22 μm, #ISEQ00010, Millipore, USA). Membranes were blocked in TBST (Tris-buffered saline with 0.1% Tween-20) containing 5% non-fat dry milk for 1 h at RT, then incubated overnight at 4 °C with primary antibodies diluted in blocking solution.

The primary antibodies include: AMPKα (1:1000, #5832, CST), phospho-AMPKα (Thr172) (1:1000, #2535, CST), mTOR (1:1000, #2983, CST), phospho-mTOR (Ser2448) (1:1000, #5536, CST), PKM1/2 (1:1000, #3190, CST), PKM2 (1:1000, #4053, CST), Arg1 (1:1000, #93668, CST, USA), PFKFB3 (1:1000, #A3934, ABclonal, China), and β-actin (1:1000, #AC026, ABclonal, China).

After three 10-min washes with TBST, membranes were incubated with HRP-conjugated goat anti-rabbit IgG (1:3000, #1706515, Bio-Rad, USA) for 1 h at RT. Protein bands were visualized using enhanced chemiluminescence substrate (ECL; #K-12045-D50, Advansta, China) and imaged on an Azure C280 system. Band intensities were quantified using ImageJ software and normalized to β-actin as a loading control.

### 2.8 Flow cytometric analysis of microglial polarization

Single-cell suspensions were prepared from the ischemic hemispheres as previously described [25]. Briefly, minced brain tissues were enzymatically dissociated using 0.25% Trypsin-EDTA (#25200056, Gibco Life Technologies, USA) and DNase I (50 U/mL, #D8071, Solarbio, China) at 37 °C for 30 min. The digestion was terminated by adding complete Dulbecco's modified Eagle medium/F12 (DMEM/F12; #11330032, Gibco Life Technologies, USA) supplemented with 10% fetal bovine serum (FBS; #A0500-3011, Cegrogen Biotech GmbH, Germany). Cell suspensions were filtered through a 70 μm cell strainer, washed with ice-cold 3% FBS-PBS, and treated with ammonium-chloride-potassium lysis buffer (150 mM NH_4_Cl, 10 mM KHCO_3_, 0.1 mM EDTA; pH 7.2) to remove red blood cells. Leukocytes were enriched by discontinuous Percoll density gradient centrifugation. The cell pellet was resuspended and layered onto a 50%, 70% Percoll gradient (#17-0891-01, GE Healthcare, USA), followed by centrifugation at 800×*g* for 25 min at 4 °C (brake off). The microglia-enriched fraction was collected from the interphase, washed three times with 3% FBS-PBS, and resuspended for staining.

For immunophenotyping, isolated cells were adjusted to 1 × 10^7^ cells/mL and stained with the following surface marker antibodies at 4 °C for 30 min in the dark: CD45 (PE-Cyanine 7; 1:80, #25-0451-82), CD11b (PE; 1:100, #12-0112-82), and F4/80 (PerCP-Cy5.5; 1:80, #45-4801-82). To assess intracellular proteins, cells were stimulated with a Cell Stimulation Cocktail (#00-4975-93) for 6 h at 37 °C under 5% CO_2_. Following stimulation, cells were fixed, permeabilized, and stained overnight at 4 °C with iNOS (FITC; 1:50, #53-5920-82) and Arg1 (APC; 1:50, #17-3697-82). All antibodies and reagents for flow cytometry were purchased from Thermo Fisher Scientific, USA. Data were acquired on a BD LSRFortessa™ flow cytometer and analyzed with FlowJo v10.8 software. Microglia were gated as live, single CD45^+^CD11b^+^F4/80^+^ cells [26]. The M1 phenotype was defined as CD45^+^CD11b^+^ iNOS^+^, and the M2 phenotype as CD45^+^CD11b^+^ Arg1^+^. The detailed gating strategy (live cells→singlets→CD45^+^→CD45^+^CD11b^+^F4/80^+med^ and CD45^+^→ CD45^+^CD11b^+^ → iNOS^+^ or Arg1^+^) is shown in Supplementary Fig. 5A.

### 2.9 Enzyme-linked immunosorbent assay (ELISA)

Concentrations of interleukin (IL)-1β, IL-6, and tumor necrosis factor (TNF)-α in cell culture supernatants or ischemic hemisphere tissue homogenates were quantified using uncoated ELISA kits according to the manufacturer's instructions. Briefly, microplates were coated with capture antibodies overnight at 4 °C. After washing, the plates were blocked for 1 h at RT. Standards and samples were then added and incubated for 2 h, followed by sequential incubation with biotinylated detection antibodies for 1 h and HRP-conjugated streptavidin for 30 min. Plates were washed five times between each step. Tetramethylbenzidine substrate was added for color development (15 min in the dark), and the reaction was terminated with stop solution. Absorbance was immediately measured at 450 nm using a SpectraMax M2 microplate reader.

Cytokine concentrations in the samples were calculated from standard curves generated for each assay. The following ELISA kits were used: Mouse IL-1β (#88-7013-22), Mouse TNF-α (#88-7324-22), and Mouse IL-6 (#88-7064-22) Uncoated ELISA Kit, all from Thermo Fisher Scientific, USA.

### 2.10 Primary microglial cell cultures

Primary microglia were isolated from postnatal day 1−3 C57BL/6 mice following established protocols [27]. Briefly, brain tissues were minced and dissociated in DMEM (#L120KJ, Shanghai Basalmedia Technologies Co., Ltd., China) containing penicillin-streptomycin (100 U/mL and 100 μg/mL, respectively). The cell suspension was filtered through a 70-μm strainer and centrifuged. The pellet was resuspended in complete DMEM supplemented with 10% FBS and recombinant mouse granulocyte-macrophage colony-stimulating factor (GM-CSF; 25 ng/mL, #51048-MNAH, Sino Biological, China) and then plated into culture flasks. The medium was changed every 3−4 days. After 10−14 days, microglia were detached by orbital shaking (1.0×*g*, 2 h, 37 °C), collected by centrifugation, and resuspended for further experiments.

Cell identity and purity were confirmed by flow cytometry. Cells were stained on ice in the dark for 30 min with the following antibodies: PE-Cy7-conjugated CD45 (1:80, #25-0451-82), PE-conjugated CD11b (1:100, #12-0112-82), and PerCP-Cy5.5-conjugated F4/80 (1:150, #45-4801-82). Microglia were identified as CD45^+^CD11b^+^ F4/80^+^ cells, and only preparations with purity ∼94% were used for subsequent experiments [28].

### 2.11 Oxygen-glucose deprivation and reoxygenation (OGD/R) model

To mimic CIRI *in vitro*, an OGD/R model was established in primary microglia based on a previously described protocol [29]. Briefly, cells were pretreated with VK (0.1–16 μg/mL) for 12 h under normal culture conditions. For OGD induction, the medium was replaced with glucose-free DMEM, and cells were incubated at 37 °C for 2 h in a hypoxic chamber containing 1% O_2_, 5% CO_2_, and 94% N_2_. Reoxygenation was initiated by replacing the medium with complete high-glucose DMEM supplemented with 10% FBS, followed by incubation under normoxic conditions for 18 h.

### 2.12 Microglia-neuron co-culture with OGD/R insult

Primary microglia were seeded in the upper inserts of Transwell plates (pore size: 0.4 μm), and primary neurons in the lower chambers of 24-well plates. After attachment, microglia were subjected to OGD/R, while neurons were pretreated with VK (2, 4, and 8 μg/mL) for 24 h. Following 2 h of OGD, fresh medium was added to both chambers, and microglia inserts were transferred to neuronal plates for 18 h reoxygenation co-culture. Supernatants from the lower chambers were collected, centrifuged (500×*g*, 10 min, 4 °C), and used for ELISA to detect IL-6, TNF-α, and IL-1β.

### 2.13 Seahorse XF cell energy metabolism analysis

Mitochondrial and glycolytic functions [30,31] were assessed using the Seahorse XFe96 Analyzer (Agilent Technologies, USA) through the mitochondrial stress test (MST) and glycolysis stress test (GST) to measure the oxygen consumption rate (OCR) and extracellular acidification rate (ECAR), respectively. Primary microglia were seeded in Seahorse XF96 cell culture microplates (102416-100, Agilent Technologies, USA) at a density of 2.5 × 10^4^ cells/well. After overnight attachment, cells were treated with VK (2, 4, and 8 μg/mL) for 24 h and subsequently subjected to the OGD/R procedure described in Section 2.11. On the day before the assay, the sensor cartridge was hydrated in XF Calibrant Solution and incubated overnight at 37 °C in a non-CO_2_ incubator. The mitochondrial modulators—including rotenone (#sc-203242), oligomycin A (#sc-201551), carbonyl cyanide-4-(trifluoromethoxy) phenylhydrazone (FCCP; #sc-203578), and antimycin A (#sc-202467)—were obtained from Santa Cruz Biotechnology (USA). For the MST, the following compounds were loaded into injection ports to achieve the indicated final concentrations per well: port A, 5 μM oligomycin A; port B, 1 μM FCCP; and port C, 1 μM rotenone/antimycin A (Rot/AA). For the GST, the ports were loaded as follows: port A, 1 μM Rot/AA; and port B, 50 mM 2-deoxy-d-glucose (2-DG; #D8375-10 MG, Sigma-Aldrich, USA).

For both assays, the culture medium was replaced with pre-warmed XF Base Medium supplemented as follows: for the MST, with 1 mM pyruvate, 2 mM glutamine, and 10 mM glucose; and for the GST, with 2 mM glutamine (pH 7.4). The cell plate was equilibrated for 45−60 min at 37 °C in a non-CO_2_ incubator before measurement. Real-time OCR and ECAR were recorded following sequential injection of modulators according to the pre-programmed Seahorse stress test protocol. Key metabolic parameters—including basal respiration, ATP production, maximal respiration, spare respiratory capacity, proton leak, basal glycolysis, glycolytic capacity, and glycolytic reserve—were calculated and analyzed using the Seahorse Wave Desktop Software (Agilent Technologies, USA).

### 2.14 Lactate production assay

Extracellular lactate accumulation was determined using a commercial Lactate Assay Kit-WST (#L256, DOJINDO LABORATORIES, Japan). Primary microglia were pretreated with VK (2, 4, and 8 μg/mL) for 24 h and then subjected to OGD/R as described above. After the reoxygenation phase, culture supernatants were collected and centrifuged (500×*g*, 10 min, 4 °C) to remove debris, then appropriately diluted. Lactate concentrations were quantified according to the manufacturer's instructions by measuring absorbance at 450 nm using a microplate reader.

### 2.15 Transmission electron microscopy (TEM) analysis of mitochondrial ultrastructure in microglia

To assess mitochondrial morphology, primary microglia subjected to the OGD/R procedure were processed for TEM. Following treatment, ischemic hemispheres or cells were detached, quenched, and immediately fixed in 2.5% glutaraldehyde (in 0.1 M PBS, pH 7.4) at 4°C for 24 h. Following aldehyde fixation, samples were post-fixed in 1% osmium tetroxide, dehydrated through a graded ethanol series, and embedded in SPI-PON 812 epoxy resin.

Ultra-thin sections (70−80 nm) were cut, collected on Formvar-coated copper grids, and double-stained with saturated uranyl acetate and lead citrate. Grids were examined using a JEM-1400Plus transmission electron microscope (JEOL Ltd., Japan) operating at 80 kV. Mitochondrial ultrastructure was evaluated for parameters [32] including matrix density, cristae integrity, and membrane continuity. Abnormal mitochondria were defined as exhibiting at least one of the following characteristics [33]: paracrystalline inclusions; linearization or angular distortion of cristae; concentric layering of cristae membranes; matrix compartmentalization or nanotunneling; or doughnut-shaped morphology.

### 2.16 Isotopic tracing with U–^13^C-glucose

To dynamically assess glycolytic flux [34], primary microglia were pretreated with or without VK for 24 h and then subjected to OGD/R. During the OGD/R period, cells were cultured in glucose-free DMEM supplemented with 2.5 mM uniformly labeled U–^13^C-glucose (#110187-42-3, Cambridge Isotope Laboratories, USA) and 10% FBS for 4 h.

After labeling, cells were rapidly washed with ice-cold PBS and metabolic activity was quenched by snap-freezing in liquid nitrogen. Cell pellets were stored at −80 °C until extraction. Metabolites were extracted by ultrasonication in 80% methanol, followed by centrifugation at 14,000×*g* at 4 °C for 15 min. The supernatant was vacuum-dried to obtain metabolite pellets. Dried samples were reconstituted in acetonitrile/water and analyzed via liquid chromatography-tandem mass spectrometry (LC-MS/MS) using an Agilent 6470 system equipped with a hydrophilic interaction liquid chromatography column (#863757-902, Agilent Technologies, USA). The mobile phase consisted of acetonitrile/water containing 10 mM ammonium acetate. Electrospray ionization (ESI) in negative mode with full-scan acquisition was used. Isotopologue distributions were quantified to evaluate real-time glycolytic flux.

### 2.17 Arginine content assay

Intracellular arginine content was determined using a commercial assay kit (#BC5635, Solarbio, China). After treatment, cells were lysed and processed according to the manufacturer's instructions. The final supernatant was derivatized in a 96-well plate, and absorbance was measured at 525 nm. Arginine concentration was calculated using the following formula: Arg (μM/cell) = 0.928 × (ΔA sample/ΔA standard), where ΔA represents the absorbance value corrected for the blank.

### 2.18 ATP content assay

Cellular ATP levels were measured using a commercial assay kit (#BC0305, Solarbio, China). Deproteinized cell lysates were mixed with assay reagents in a 96-well plate. Absorbance was immediately measured at 340 nm (A_1_). After 3 min of incubation at 37 °C, absorbance was measured again (A_2_). ATP concentration was calculated as: ATP (μM per cell) = 0.625 × (ΔA sample/ΔA standard), where ΔA = A_1_–A_2_.

### 2.19 Nicotinamide adenine dinucleotide (NAD^+^)/NADH assay

Levels of oxidized (NAD^+^) and reduced (NADH) were quantified using a commercial assay kit (#S0175, Beyotime, China). Cell lysates were split: one aliquot was used for total NAD (NAD^+^ + NADH) measurement, and the other was heated to decompose NAD^+^ for NADH-specific measurement. Both samples were incubated with alcohol dehydrogenase and a chromogen, and absorbance was measured at 450 nm. NAD^+^ concentration and the NAD^+^/NADH ratio were calculated according to the manufacturer's protocol.

### 2.20 2-NBDG glucose uptake assay

Glucose uptake in primary microglia was quantified using the fluorescent glucose analog 2-[N-(7-Nitrobenz-2-oxa-1,3-diazol-4-yl) amino]-2-deoxy-d-glucose (2-NBDG). After pretreatment and OGD/R challenge, cells were washed and then incubated with 100 μM 2-NBDG (#52782ES03, Yeasen Biotechnology Co., Ltd., Shanghai, China) at 37 °C for 30 min in the dark. After probe removal and washing, fluorescence images were captured immediately on a fluorescence microscope (#Axiovert 5, ZEISS, Germany) with excitation/emission wavelengths of 488/542 nm. For each group, images from ≥3 random fields per experiment (n = 3 independent experiments) were semi-quantified using ImageJ software.

### 2.21 MitoSOX™ red assay

Mitochondrial superoxide production was detected using the MitoSOX™ Red fluorescent probe (#S0061S, Beyotime Biotechnology, China). For primary microglia subjected to drug treatment and OGD/R conditions, the culture medium was removed, and cells were washed with pre-warmed PBS (pH 7.4). Where indicated, cells were co-incubated with a mitochondrial-specific dye (#HB220418, Yeasen Biotechnology Co., Ltd, China) to confirm probe localization. Cells were then incubated with 5 μM MitoSOX™ Red working solution at 37 °C in a 5% CO_2_ incubator for 30 min in the dark. Following incubation, cells were processed for two parallel detection methods:

*Fluorescence microscopy* After probe removal and washing, cells were immediately visualized and imaged using a fluorescence microscope (#Axiovert 5, ZEISS, Germany) at excitation/emission wavelengths of 510/580 nm. For each group, images from at least five randomly selected fields were obtained, and the experiment was independently repeated three times.

*Flow cytometry* After staining, cells were detached, washed, and resuspended in PBS containing 2% FBS. Fluorescence was acquired on a BD LSRFortessa™ flow cytometer, and data were analyzed using FlowJo v10.8 software to determine the percentage of MitoSOX™ Red-positive cells and the mean fluorescence intensity.

### 2.22 Mitochondrial membrane potential (JC-1) assay

Primary microglia were pretreated with VK (2,4 μg/mL), subjected to OGD/R, and then stained with JC-1 (5 μg/mL, 20 min) kit (#M8650, Solarbio, China). carbonyl cyanide m-chlorophenyl hydrazone(CCCP, 10 μM, 20 min), an uncoupler of oxidative phosphorylation, was used as a positive control to induce complete mitochondrial depolarization. JC-1 monomers (depolarized mitochondria) and aggregates (polarized) were detected by fluorescence microscopy (Axiovert 5 digital, ZEISS) and flow cytometry (NovoCyte Advanteon, Agilent Technologies, USA). Quantification of JC-1 monomer-positive cells was performed using FlowJo v10.8 software. Excitation/Emission (EX/EM) wavelengths: 490/530 nm for JC-1 monomers; 525/590 nm for JC-1 aggregates.

### 2.23 Glycolysis and OXPHOS assay

Primary microglia were seeded in 24-well plates for lactate measurement or in white 96-well plates for ATP measurement. After OGD/R, cells were treated for 12 h under four conditions: control (standard medium), VK (standard medium with VK), arginine deprivation (arginine-free medium with dialyzed FBS), or arginine deprivation plus VK. Arginine-free medium consisted of arginine-deficient DMEM supplemented with dialyzed FBS. After treatment, the medium was removed. To assess glycolytic capacity, cells were incubated with or without oligomycin (1.25 μM) for 3–5 h at 37 °C with 5 % CO_2_. For metabolic pathway dependency, separate wells were treated with oligomycin (1.25 μM), 2-DG (25 mM), or vehicle for 5 h according to the manufacturer's protocol (Glycolysis/OXPHOS Assay Kit, #G270, DOJINDO LABORATORIES, Japan).

After inhibitor treatment, culture supernatants were collected for lactate measurement. Briefly, 20 μL of supernatant was diluted 10-fold with ultrapure water, mixed with 80 μL of lactate working solution, and incubated at 37 °C for 30 min in the dark. Absorbance was read at 450 nm. For ATP measurement, 100 μL of ATP working solution was added directly to each well of the white 96-well plate. The plate was shaken for 2 min, incubated at 25 °C for 10 min, and relative light units (RLU) were measured using a luminometer.

### 2.24 Quantitative real-time polymerase chain reaction (qRT-PCR) analysis

Primary microglia were pretreated with VK (2, 4, and 8 μg/mL) for 24 h, and then subjected to OGD/R insult. Total RNA was extracted using a commercial RNA isolation kit (#AG21022, Accurate Biotechnology Co., Ltd, China), and complementary DNA (cDNA) was synthesized using a reverse transcription kit (#RR037A, Takara, Japan). PCR amplification was performed using gene-specific primers (Table 1), SYBR Green Master Mix, and a quantitative PCR instrument. The amplification conditions were as follows: initial denaturation at 95 °C for 30 s, followed by 40 cycles of 95 °C for 5 s and 60 °C for 30 s. β-actin served as the reference gene for normalization, and relative gene expression was calculated using the 2^−ΔΔCT^ method.

**Table 1:** Primer sequences.

Species	Gene	Sequence
Mouse	arg*1*	5′-CTCCAAGCCAAAGTCCTTAGAG-3′
		5′-AGGAGCTGTCATTAGGGACATC-3′
Mouse	*asl*	5′-CTATGACCGGCATCTGTGGAA-3′
		5′-AGCAACCTTGTCCAACCCTTG-3′
Mouse	*ass1*	5′-ACACCTCCTGCATCCTCGT-3′
		5′-GCTCACATCCTCAATGAACACCT-3′
Mouse	*il-1β*	5′-GCAACTGTTCCTGAACTCAACT-3′
		5′-ATCTTTTGGGGTCCGTCAACT-3′
Mouse	*il-6*	5′-TAGTCCTTCCTACCCCAATTTCC-3′
		5′-TTGGTCCTTAGCCACTCCTTC-3′
Mouse	*tnf*	5′-CCCTCACACTCAGATCATCTTCT-3′
		5′-GCTACGACGTGGGCTACAG-3′
Mouse	*pfk1*	5′-TGACCTTAGTGAACCACGGC-3′
		5′-GGACTTGGGGCAGGCTTAAT-3′
Mouse	*ogdh*	5′-GTTTCTTCAAACGTGGGGTTCT-3′
		5′-GCATGATTCCAGGGGTCTCAAA-3′
Mouse	*pkm2*	5′-GCCGCCTGGACATTGACTC-3′
		5′-CCATGAGAGAAATTCAGCCGAG-3′
Mouse	*pfkfb3*	5′-CCCAGAGCCGGGTACAGAA-3′
		5′-GGGGAGTTGGTCAGCTTCG-3′
Mouse	*idh1*	5′-ATGCAAGGAGATGAAATGACACG -3′
		5′-GCATCACGATTCTCTATGCCTAA-3′
Mouse	*idh2*	5′-GGAGAAGCCGGTAGTGGAGAT-3′
		5′-GGTCTGGTCACGGTTTGGAA-3′
Mouse	*inos*	5′-GTTCTCAGCCCAACAATACAAGA-3′
		5′-GTGGACGGGTCGATGTCAC-3′

### 2.25 Silencing of AMPKα in BV2 cells

To achieve AMPKα knock down [35], BV2 cells were transfected with a specific small interfering RNA (siRNA) targeting the *PRKAA1* gene (si-AMPKα) or a non-targeting negative control siRNA (si-Control). BV2 cells were obtained from the Cell Resource Center of the Institute of Basic Medical Sciences, Chinese Academy of Medical Sciences, with resource number 1101MOU-PUMC000063. Both siRNAs were synthesized by GenePharma Corporation (Shanghai, China). The si-AMPKα duplex consisted of the sense strand 5′-CAAGUCGACCAAAUGAUAUU-3′ and the antisense strand 5′-UAUCAUUUGGUCGACUUUGUU-3′. The negative control siRNA sequences were 5′-UUCUCCGAACGUGUCACGUTT-3' (sense) and 5′-ACGUGACACGUUCGGA GAATT-3′ (antisense).

Transfection was performed using Lipofectamine™ 3000 transfection (#L3000008, Invitrogen, USA) with 50 nM siRNA according to the manufacturer's protocol. After 6 h, the transfection mixture was replaced with fresh complete culture medium, and cells were cultured for an additional 48 h to allow efficient gene silencing. Knockdown efficiency was confirmed by Western blotting before functional assays.

### 2.26 Untargeted metabolomics profiling

Untargeted metabolomic profiling was performed on the ischemic hemisphere. Briefly, tissues were pulverized in liquid nitrogen and homogenized in ice-cold 80% methanol. After centrifugation (15,000×*g*, 20 min, 4 °C), supernatants were diluted and re-centrifuged before LC-MS/MS analysis. The resulting supernatant was analyzed by LC-MS/MS. Quality control (QC) samples were prepared by pooling aliquots from all samples.

Chromatographic separation was performed on a Hypersil GOLD C18 column (2.1 × 100 mm, 1.9 μm; Thermo Scientific, USA) maintained at 40 °C with a flow rate of 0.2 mL/min, using water with 0.1% formic acid (A) and methanol (B) as mobile phases. Mass spectrometry analysis was performed with ESI in positive/negative switching modes, full-scan range *m*/*z* 100−1500 (resolution 70,000), and data-dependent MS/MS acquisition.

Raw data were processed using XCMS (v3.12.0) for feature detection and alignment. Features were filtered (blank subtraction and QC coefficient of variation <30%) and normalized using QC-based robust LOESS correction. Metabolites were annotated using the Human Metabolome Database (v4.0), Kyoto Encyclopedia of Genes and Genomes (KEGG; release 107.0), and LIPID MAPS (v3.0) databases (mass tolerance 10 ppm) [36]. Differential metabolites were identified using the Wekemo Bioincloud (https://www.bioincloud.tech), with significance defined as VIP>1.0 (from OPLS DA) and *P* < 0.05 (Student's t-test).

### 2.27 Proteomic analysis

Brain tissues were lysed in SDT buffer (4% SDS, 100 mM Tris-HCl, pH 7.6) containing protease inhibitors. Protein concentrations were determined using the BCA assay. After quality assessment by SDS-PAGE and Coomassie blue staining, proteins from all specimens were pooled to generate a quality control sample. Proteins were digested using a filter-aided sample preparation protocol with sequencing-grade trypsin (1:50, w/w) at 37 °C for 16 h. Peptides were desalted on C18 cartridges (Waters), lyophilized, and reconstituted in 0.1% formic acid. Concentration was measured by OD_280_ before the addition of indexed retention time standards (Biognosys, Switzerland).

Data-independent acquisition (DIA) mass spectrometry was performed on a Vanquish Neo UHPLC system coupled to an Orbitrap Astral mass spectrometer (Thermo Scientific, USA). Peptides were separated on a 50-cm C18 column (75-μm inner diameter, 1.6-μm particle size) with a 90-min gradient (5%−28% acetonitrile/0.1% formic acid). MS parameters were: MS1 scan range 380−980 *m*/*z* (resolution 240,000); MS2 acquisition with 299 variable windows (2 *m*/*z* isolation width); and normalized collision energy of 25 eV.

Raw DIA data were processed using DIA-NN (v1.8.1) in library-free mode, with carbamidomethylation as a fixed modification and oxidation/N-terminal acetylation as variable modifications. Protein quantification was performed at the MS1 level with interference correction [36]. Statistical analyses were completed using the Wekemo Bioincloud (https://www.bioincloud.tech). Differentially expressed proteins were identified with a threshold of |log_2_ (Fold Change) |>1.5 and an adjusted *P* < 0.05 (Student's t-test with Benjamini-Hochberg correction).

### 2.28 Single-cell RNA sequencing (scRNA-seq) and data analysis

After the final treatment, ischemic cerebral cortex tissues were rapidly dissected, snap-frozen, and sent for scRNA-seq [37]. Single-cell suspensions were prepared, and library construction was performed by GENEPIONEER Technology Co., Ltd. Rigorous QC ensured cell viability ≥85%, a concentration of 700−1200 cells/μL, and a cell diameter of 5−40 μm. Libraries were constructed using the MobiNova high-throughput single-cell platform based on Gel Bead-in-Emulsion (GEM) technology.

Sequencing was conducted on an Illumina NovaSeq 6000 platform (Illumina, USA) using paired-end 150 bp reads, targeting a depth of 3−15 million reads per cell. Data were processed using Mobivision software (v3.2; MobiDrop Technology, China) and the Seurat package (v4.0.0) in R. The workflow included QC, alignment to the GRCm38/mm10 reference genome, and construction of a gene expression matrix. Unsupervised clustering, identification of differentially expressed genes, and functional enrichment analysis (Gene Ontology [GO] and KEGG pathways) were carried out. Dimensionality reduction and visualization were performed using principal component analysis (PCA), t-distributed stochastic neighbor embedding, and uniform manifold approximation and projection.

### 2.29 Statistical analysis

Statistical analyses were performed using GraphPad Prism 10.4.0 (GraphPad Software, USA) and R 4.1.2 (with Python 3.9 for multi-omics data). Sample sizes were determined from pilot studies and prior literature to ensure adequate statistical power. All animal experiments were randomized using a random number table for group allocation and were blinded to investigators during outcome assessment (e.g., neurological scoring, histological quantification). Cell-based experiments were independently repeated at least three times, with technical triplicates per biological repeat.

Data distribution and variance homogeneity were assessed using the Kolmogorov-Smirnov test and Levene's test, respectively. Based on these assessments, the following tests were applied:

For normally distributed data with homogeneous variance:1) Single-factor comparisons (e.g., multiple VK concentrations vs control) were analyzed by one-way analysis of variance (ANOVA) followed by Dunnett's post hoc test for comparisons with the vehicle or OGD/R group; 2) Two-factor comparisons (e.g., treatment x time point) were analyzed by two-way ANOVA followed by Bonferroni's post hoc test; 3) Pairwise comparisons between two groups (e.g., sham vs vehicle) were performed using an unpaired, two-tailed Student's t-test.

For non-normally distributed data with heterogeneous variance: Kruskal-Wallis test was used for multiple groups, followed by Dunn's post hoc test with Bonferroni correction.

For multi-omics data, differential features (metabolites, proteins, genes) were identified using linear models with empirical Bayes moderation (limma package in R). Significance was defined as |log_2_ (Fold Change) |>1 and adjusted *P* < 0.05 (Benjamini-Hochberg false discovery rate correction).

All tests were two-tailed, and *P* < 0.05 was considered statistically significant. Effect sizes are reported for major findings.

## 3 Results

### 3.1 VK attenuates neuroinflammation and reshapes the cerebral cellular landscape after stroke

**Fig. 1:**
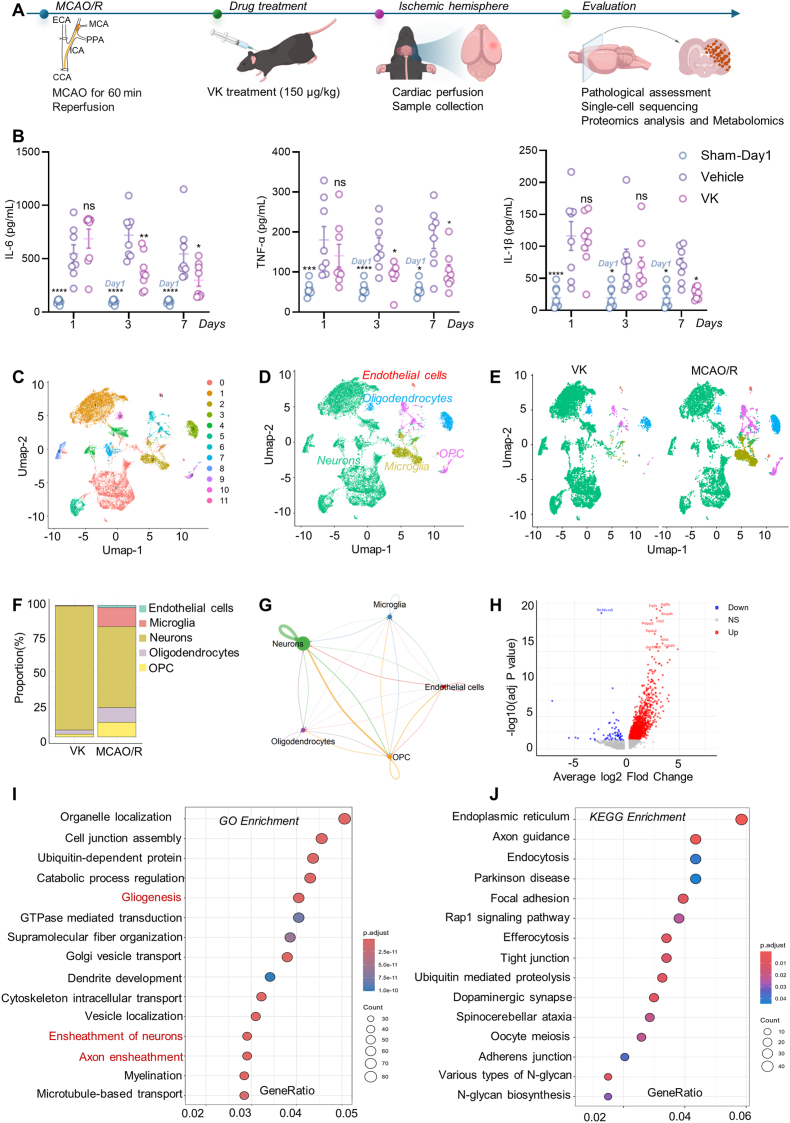
Vespakinin-M (VK) attenuates neuroinflammation and reshapes cerebral cellular landscapes after ischemic stroke in mice. (A) Schematic illustration of the experimental timeline showing the procedure for middle cerebral artery occlusion/reperfusion (MCAO/R) procedure, VK administration (*i.p.)* injection, 150 μg/kg/day starting within 1 h post-reperfusion for 7 consecutive days), and tissue collection at indicated time points; (B) The release of interleukin (IL)-6, tumor necrosis factor-alpha (TNF-α), and IL-1 beta (β) in the ischemic hemisphere at indicated days after MCAO/R (n = 8 mice/group).Note: Sham group was measured only on day 1; its values on days 3 and 7 are the day 1 data repeated for visual reference (not independent sampling.Data are presented as means ± SEM. Data were analyzed by two-way ANOVA followed by Bonferroni's post hoc test. ∗*P* < 0.05, ∗∗*P* < 0.01, ∗∗∗*P* < 0.001, ∗∗∗∗*P* < 0.0001 vs Vehicle group at the same time point; ns, not significant; (C) Uniform Manifold Approximation and Projection (UMAP) plot of single-cell RNA sequencing (scRNA-seq) data showing 11 transcriptomically distinct cell clusters from the ischemic hemisphere; (D) Annotation of the five major cell lineages (endothelial cells, microglia, neurons, oligodendrocytes, oligodendrocyte precursor cells) identified in panel (C); (E) UMAP visualization comparing cellular landscapes between the MCAO/R and VK-treated groups; (F) Quantification of relative abundances of major cell types following VK treatment; (G) Circos plot depicting the ligand-receptor-mediated intercellular communication networks, with edge thickness representing interaction strength; (H) Volcano plot showing differentially expressed genes (DEGs) in microglia from VK versus MCAO/R groups (|log_2_ (fold change) |>1, adjusted *P* < 0.05); (I) Gene Ontology (GO) biological process enrichment analysis of microglial DEGs; (J) Significantly enriched Kyoto Encyclopedia of Genes and Genomes pathways identified from the microglial DEG analysis.

While previous studies have confirmed that VK reduces cerebral infarct volume, its impact on the dynamic neuroinflammatory landscape remained unclear. The present study further validated this effect in an MCAO/R mouse model (Supplementary Fig. 1A and B; as described in Supplementary Methods 1.1). To address this, MCAO/R mice were treated with VK (150 μg/kg/day, i.p., for 7 days starting 1 h post-reperfusion) and the brain tissues were analyzed across multiple timepoints (Fig. 1A). We first confirmed that VK significantly reduced infarct volume in the present cohort (Supplementary Fig. 1A and 1B). Moreover, VK treatment markedly alleviated oxidative stress in the ischemic hemisphere, as evidenced by increased superoxide dismutase (SOD) activity and decreased levels of malondialdehyde (MDA) and lipid hydroperoxide (LPO) (Supplementary Fig. 1C; Supplementary Methods 1.2). Regarding glutathione peroxidase (GSH-PX) activity, VK-treated mice exhibited a trend towards elevation compared with the vehicle, although this did not reach statistical significance (Supplementary Fig. 1C). These results confirm that VK confers robust protection against both structural damage and redox imbalance after stroke.

VK modulated the levels of pro-inflammatory cytokines in the ischemic hemisphere. On day 1 post-MCAO/R, the release of IL-6, TNF-α, and IL-1β was markedly elevated in both the VK and vehicle groups compared with the sham group (Fig. 1B), suggesting that acute-phase inflammation was not suppressed. This phenomenon may reflect the physiological necessity of early inflammatory responses in tissue clearance and the initiation of repair [38]. By day 3, VK selectively attenuated IL-6 release (TNF-α and IL-1β remained unchanged compared with vehicle), indicating an early, cytokine-specific effect [39]. Comparably, VK suppressed all three cytokines by day 7 (Fig. 1B).

To define the cellular underpinnings of this modulation, we performed scRNA-seq on the ischemic hemisphere. Unsupervised clustering identified 12 transcriptionally distinct populations (Fig. 1C), which were annotated as five major lineages: endothelial cells, microglia, neurons, oligodendrocytes, and oligodendrocyte precursor cells (Fig. 1D). Comparative analysis showed that VK treatment markedly altered the cellular repertoire, decreasing the proportion of microglia while increasing that of neurons (Fig. 1E and F). This shift suggests that VK curbs microglia-driven inflammatory responses and promotes a pro-survival microenvironment. Cell-cell interaction analysis further confirmed extensive intercellular communication, with microglia serving as a central signalling node (Fig. 1G).

To dissect VK's direct effect on microglia, we analyzed differential gene expression within the microglial cluster. A volcano plot identified a distinct transcriptional profile in VK-treated microglia (significantly altered genes defined as |log_2_ (fold change) |>1 and adjusted *P* < 0.05) (Fig. 1H). VK treatment led to a marked preponderance of upregulated genes (e.g., *Ugt8a*, *Fa2h*, *Pstpip2*), which drive multiple protective pathways such as sphingolipid metabolism, over the downregulation of specific targets (e.g., *Cacna1a*, *Meg3*, *Rn18s-rs5*) that inhibit discrete damaging signals (Supplementary Fig. 2). GO enrichment highlighted processes including organelle localization, cell junction assembly, and ubiquitin-dependent protein catabolism (Fig. 1I). Consistently, KEGG analysis implicated pathways in endoplasmic reticulum protein processing, axon guidance, and endocytosis (Fig. 1J). Collectively, these data indicate that VK mitigates neuroinflammation primarily by orchestrating microglial transcriptional reprogramming toward a state conducive to protein homeostasis and neural repair.

### 3.2 VK modulates IBA1^+^ microglial accumulation and inhibits inflammatory responses after CIRI, contributing to its neuroprotective effects

**Fig. 2:**
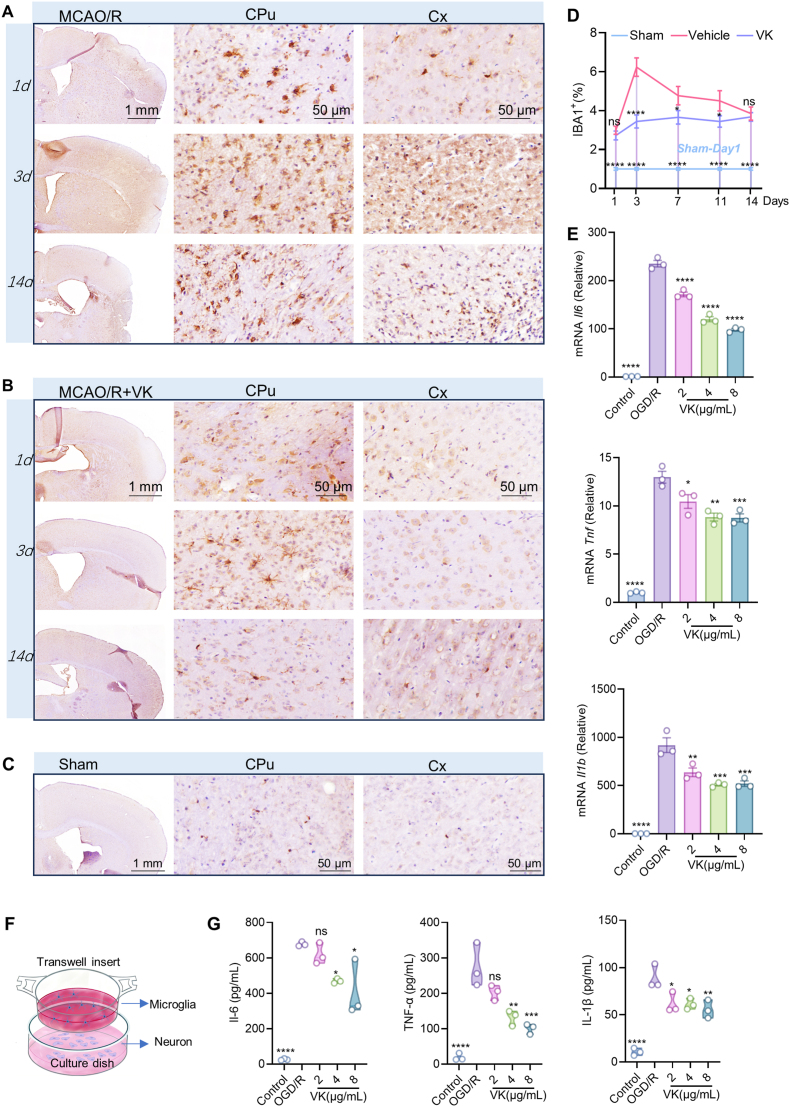
VK modulates IBA1^+^ microglial accumulation and inhibits inflammatory responses after CIRI, contributing to its neuroprotective effects. (A–C) Representative immunohistochemical images of IBA1^+^ microglia in caudate putamen (CPu), cerebral cortex (Cx), and striatum at indicated days post-MCAO/R. Scale bar: 1 mm (overview), 50 μm (inset); (D) Quantification of IBA1^+^ microglial density in the peri-infarct region corresponding to panels (A–C);Note: Sham values on days 3, 7, 11, and 14 are the day 1 data repeated for visual reference (not independent sampling).Data are presented as means ± SEM (n = 5 mice per group); two-way ANOVA with Bonferroni's post hoc test: ∗*P* < 0.05, ∗∗*P* < 0.01 vs MCAO/R group at the same time point; (E) Relative mRNA expression of *Il1b*, *Il6*, and *Tnf* in primary microglia subjected to oxygen-glucose deprivation/reoxygenation (OGD/R) with or without VK pretreatment, measured by RT-PCR and normalized to β-actin; (F) Schematic diagram of microglia-neuron co-culture using Transwell inserts (microglia in upper chamber, neurons in lower chamber); (G) Concentrations of pro-inflammatory cytokines (IL-6, TNF-α, and IL-1β) in the supernatant of co-cultures post-OGD/R, measured using ELISA Data in (E, G) are mean ± SEM (n = 3 independent experiments); one-way ANOVA with Tukey's post hoc test: ∗*P* < 0.05, ∗∗*P* < 0.01, ∗∗∗*P* < 0.001, and ∗∗∗∗*P* < 0.001 vs OGD/R group.

Immunohistochemical analysis using the myeloid cell marker IBA1 revealed that MCAO/R insult induced IBA1^+^ cells to transition from a ramified morphology toward an amoeboid phenotype. This alteration was attenuated by VK treatment (Fig. 2A−C; Supplementary Fig. 3). Quantitative analysis of IBA1^+^ cell density in the peri-infarct region revealed distinct temporal dynamics across different phases of CIRI (Fig. 2D). Compared with the sham group, the vehicle group displayed a significant increase in IBA1^+^ cell density at all time points. On day 1 post-MCAO/R, no significant difference in IBA1^+^ cell density was observed between the vehicle and VK-treated groups, suggesting that VK does not affect the initial accumulation of microglia in the acute phase. However, during the peak inflammatory phase (days 3, 7, and 11), VK treatment significantly reduced IBA1^+^ cell density compared to the vehicle group, indicating that VK attenuates the sustained accumulation of IBA1^+^ microglia in the peri-infarct region. By the resolution phase (day 14), IBA1^+^ cell density in both groups returned to comparable levels, with no significant difference between vehicle and VK-treated mice.

We next isolated primary microglia (purity ∼94%; Supplementary Fig. 4A) to dissect the cell-autonomous effects of VK. VK exposure (128−256 μg/mL) for 24−72 h reduced cell viability. Dose-response curve fitting yielded IC_50_ values of 129.80 μg/mL (R^2^ = 0.9866) at 24 h, 112.30 μg/mL (R^2^ = 0.9813) at 48 h, and 84.81 μg/mL (R^2^ = 0.9956) at 72 h (Supplementary Fig. 4B−D; as determined by CCK-8 assay, see Supplementary Methods 1.3). Based on this, non-cytotoxic concentrations (1−8 μg/mL) were used. VK dose-dependently restored cell viability after OGD/R insult (Supplementary Fig. 4E) and significantly suppressed the OGD/R-induced mRNA upregulation of *Il1b*, *Il6*, and *Tnf* (Fig. 2E).

To assess indirect neuroprotection, we employed a microglia–neuron co-culture system to better mimic the brain microenvironment (Fig. 2F). OGD/R induced a significant increase in pro-inflammatory cytokines in the neuronal chamber supernatant, which was markedly attenuated when microglia were pretreated with VK (Fig. 2G). This indicates that VK protects neurons, at least in part, by suppressing microglial pro-inflammatory activation.

### 3.3 VK modulates microglial polarization towards an M2-polarized phenotype after MCAO/R in mice

**Fig. 3:**
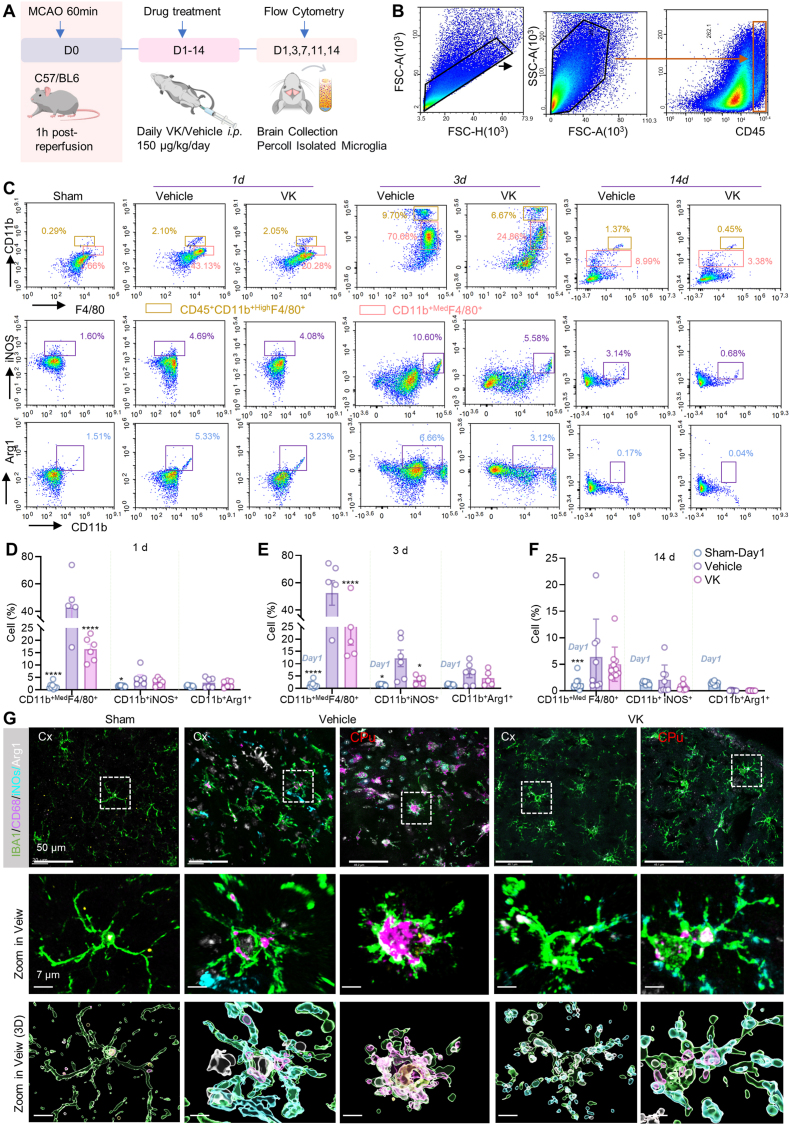
VK modulates microglial polarization after MCAO/R in mice. (A) Schematic of the experimental timeline; (B–C) Gating strategy for flow cytometric analysis of microglial phenotypes, detailed in Supplementary Fig. 5. Microglia were identified by sequential gating on live, single cells, followed by selection of CD45^+^ immune cells, and then the CD45^+^CD11b^+^F4/80^+^ population. M1 and M2 subsets were defined as CD45^+^CD11b^+^iNOS^+^ and CD45^+^CD11b^+^Arg1^+^ cells, respectively; (C) Representative flow cytometry plots showing M1 (iNOS^+^) and M2 (Arg1^+^) microglial subsets in the ischemic hemisphere at the indicated days post-MCAO/R; (D–F) Quantification of total activated microglia/macrophages (CD45^+^CD11b^+^F4/80^+^ᵐ^ed^), M1-polarized microglia (iNOS^+^), and M2-polarized microglia (Arg1^+^) at days 1 (D), 3 (E), and 7 (F) post-MCAO/R. Note: Sham group was measured only on day 1; its values on days 3 and 14 are the day 1 data repeated for visual reference (not independent sampling).(G) Representative immunofluorescence images of IBA1^+^ (microglia), CD68 (phagocytic activation marker), iNOS^+^ (M1), and Arg1^+^ (M2) cells in the ischemic hemisphere at 7 days post-MCAO/R. Scale bars: 50 μm (overview), 7 μm (insets). Data are mean ± SEM (n = 6–10 mice per group); two-way ANOVA with Bonferroni's post hoc test: ∗*P* < 0.05, ∗∗*P* < 0.01, ∗∗∗*P* < 0.001, ∗∗∗∗*P* < 0.0001 vs MCAO/R group at the same time point.

To assess the impact of VK on microglial polarization, we performed flow cytometric analysis on immune cells isolated from the ischemic hemisphere (Fig. 3A). A representative flow cytometry strategy was employed to identify CD45^+^ cells in ischemic brain tissue (Fig. 3B). Within the CD45^+^ population, we focused on CD45^+^CD11b^+^F4/80^+med^ cells—a heterogeneous population that includes both activated resident microglia (CD45^+med^CD11b^+^) and infiltrating macrophages (CD45^+hi^CD11b^+^) [40,41], here collectively termed microglia/macrophages.

Compared with the sham group, MCAO/R induced a significant increase in the proportion of CD45^+^CD11b^+^F4/80^+med^ cells (days 1, 3, 7, 11, and 14; Fig. 3 3C–F), peaking at day 3 post-MCAO/R (Fig. 3E). VK treatment reduced this increase on days 1, 3, 7, and 11. However, there were no significant differences between the VK and vehicle groups on day 14 (Fig. 3C–F; Supplementary Fig. 5B–D).

Phenotypic analysis of polarization revealed distinct dynamics in the M1 and M2 subsets. Compared with the sham group, MCAO/R group showed a significant increase in the proportion of M1-polarized cells (CD45^+^CD11b^+^iNOS^+^) on days 1, 3, 7, and 11, which returned toward baseline levels by day 14. Comparably, VK treatment reduced the proportion of M1-polarized cells on days 3 (Fig. 3C–E); on days 1, 7, and 11, a trend toward reduction was noted but did not reach statistical significance (Fig. 3C–F; Supplementary Fig. 5B–D). In contrast, the vehicle group showed a modest, non-significant increase in the proportion of M2-polarized cells (CD45^+^CD11b^+^Arg1^+^) on days 3 and 7 compared with the sham group. Notably, compared with the vehicle group, VK treatment elevated the M2 cell proportion on day 7, with a similar upward trend observed on day 11 (Fig. 3E; Supplementary Fig. 5B and 5C). Given that the CD45^+^CD11b^+^F4/80^+med^ cell population exhibited less distinct clustering at day 7, which could compromise the reliability of flow cytometric gating. We measured iNOS and Arg1 protein levels in the ischemic hemisphere by ELISA at day 7, following the procedure described in Supplementary Methods 1.4. Consistent with the flow cytometry trend, VK treatment significantly increased Arg1 expression, whereas no significant difference in iNOS levels was observed between the VK and vehicle groups (Supplementary Fig. 5E and 5F).

By day 14 post-MCAO/R, the proportion of CD45^+^CD11b^+^F4/80^+med^ cells in both the vehicle and VK groups had decreased compared with their respective earlier peaks (days 1–11), indicating resolution of the acute myeloid response (Fig. 3C–F). The IBA1^+^ cell density data (Fig. 2D) showed a similar trend. M1 microglia are closely associated with T helper 1-primed CD4^+^ T cells, whereas M2 microglia preferentially interact with Th2-primed CD4^+^ T cells [42]. Compared with the MCAO/R group, VK treatment decreased the ratio of CD3^+^CD4^+^IFN-γ^+^ cells and increased the ratio of CD3^+^CD4^+^IL-4^+^ cells (Supplementary Fig. 6).

To spatially validate these findings, we performed immunofluorescence co-staining of brain sections at day 7. Sections were stained for the microglial marker IBA1, the M1 marker iNOS, the M2 marker Arg1, and the activation marker CD68. Consistent with flow cytometry, VK significantly inhibited M1 polarization, as indicated by reduced co-localization of IBA1 with the M1 marker iNOS. Conversely, VK promoted a phenotypic switch of microglia toward the M2 subtype, as evidenced by a marked increase in the number of IBA1^+^Arg1^+^ (M2-polarized) microglia (Fig. 3G). Collectively, these results demonstrate that VK shifts the microglial polarization balance by suppressing the M1 phenotype and promoting an M2-associated state.

### 3.4 VK reprograms microglial metabolism from aerobic glycolysis to OXPHOS

CIRI drives fundamental metabolic reprogramming in microglia, shifting their primary energy production from OXPHOS to aerobic glycolysis [43,44]. To elucidate the effects of VK on microglial energy metabolism under OGD/R stress, we performed real-time extracellular flux analysis to simultaneously measure OCR (reflecting mitochondrial respiration) and ECAR (reflecting glycolytic flux).

**Fig. 4:**
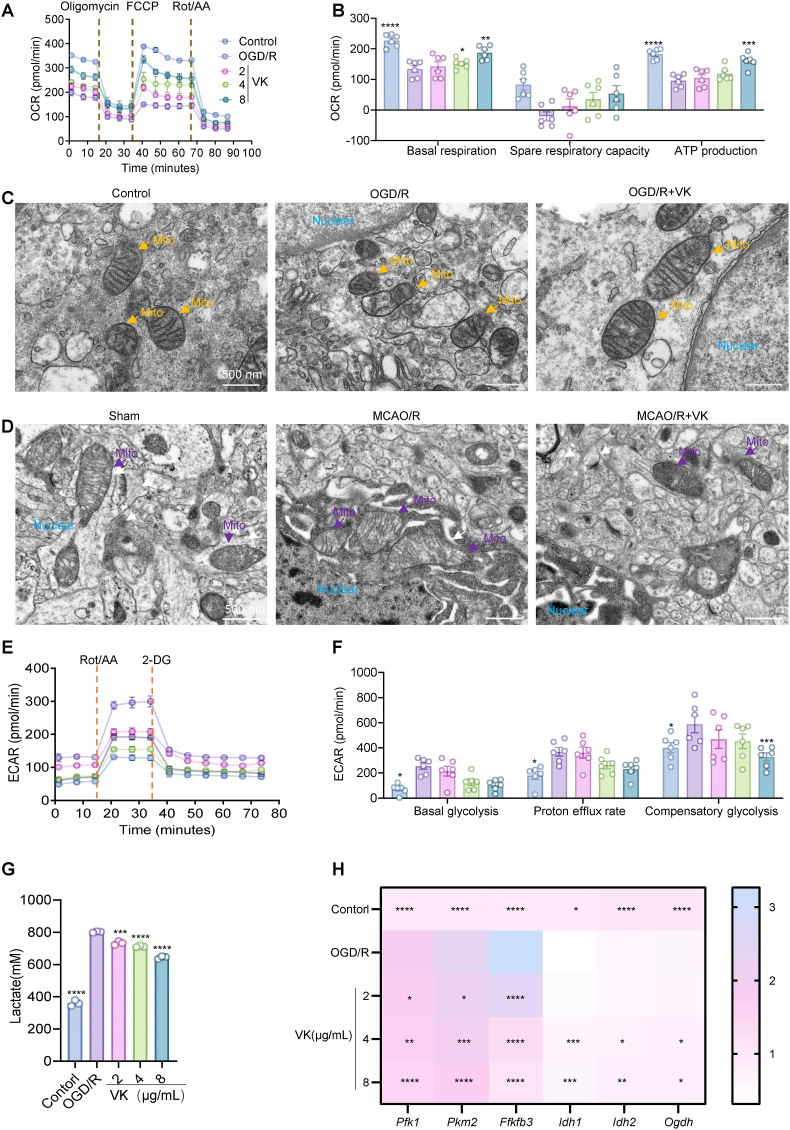
VK reprograms microglial metabolism from aerobic glycolysis to oxidative phosphorylation. (A) Mitochondrial stress test profiles showing oxygen consumption rate (OCR) in primary microglia (Control, OGD/R, OGD/R + VK [2, 4, and 8 μg/mL]). Arrows indicate sequential injections of oligomycin (Oligomycin A, 5 μM), carbonyl cyanide-4-(trifluoromethoxy) phenylhydrazone (FCCP, 1 μM), and rotenone/antimycin A (Rot/AA, 1 μM each); (B) Quantification of key mitochondrial parameters from (A), including basal respiration, adenosine triphosphate (ATP) production, maximal respiration, and spare respiratory capacity; Note that VK-treated cells exhibited a steeper OCR drop after oligomycin injection compared to OGD/R insult, indicating restored dependency on OXPHOS for ATP production. (C) Representative transmission electron microscopy images showing mitochondrial ultrastructure in primary microglia after OGD/R ± VK (8 μg/mL) treatment. Scale bar: 1 μm; (D) Representative TEM images showing mitochondrial morphology in the ischemic hemisphere at day 7 post-MCAO/R. Scale bar: 500 nm; (E) Glycolysis stress test profiles showing extracellular acidification rate (ECAR) in primary microglia. Arrows indicate sequential injections of glucose (10 mM), Oligo (5 μM), and 2-deoxy-d-glucose (2-DG, 50 mM); (F) Quantification of glycolytic parameters from (E), including glycolysis, glycolytic capacity, and glycolytic reserve; (G) Extracellular lactate concentration in culture supernatants measured by lactate assay kit; (H) Relative mRNA expression of glycolytic genes (*pfk1*, *pkm2*, *pfkfb3*), and OXPHOS-related genes (*idh1*, *idh2*, *ogdh*) in OGD/R-challenged microglia with or without VK treatment (2,4, and 8 μg/mL), measured by qRT-PCR. Data are mean ± SEM (In B and F, n = 6 independent experiments; In G and H, n = 3 independent experiments); one-way ANOVA with Tukey's post hoc test: ∗*P* < 0.05, ∗∗*P* < 0.01, ∗∗∗*P* < 0.001, ∗∗∗∗*P* < 0.0001 vs OGD/R group.

OGD/R severely impaired mitochondrial respiratory function, as evidenced by markedly reduced basal OCR, diminished ATP production, and nearly exhausted spare respiratory capacity (Fig. 4A and B). VK treatment dose-dependently restored mitochondrial respiration, with the most pronounced recovery at 8 μg/mL, which significantly rescued spare respiratory capacity (Fig. 4A andB). Notably, VK-treated groups exhibited a steeper OCR drop immediately after oligomycin injection than the OGD/R group, indicating greater coupling of mitochondrial respiration to ATP production and thus restored OXPHOS dependency.

TEM analysis further confirmed that both *in vitro* OGD/R and *in vivo* MCAO/R induced severe mitochondrial damage. In primary microglia, OGD/R exposure resulted in swollen and deformed mitochondria, fragmented and disorganized cristae, matrix vacuolization, and reduced electron density (Fig. 4C). In brain tissue, MCAO/R induced mitochondrial swelling, extensive cristae fragmentation or even loss, and liquefactive changes in the matrix (Fig. 4D). These structural disruptions were markedly ameliorated by VK pretreatment, which effectively preserved mitochondrial morphological integrity, attenuated cristae fragmentation and swelling, and prevented structural damages associated with functional impairments (Fig. 4C and D).

Concurrently, OGD/R triggered a compensatory increase in glycolysis, as evidenced by elevated ECAR. While low VK concentrations (2 μg/mL) had minimal effect, higher concentrations (4, 8 μg/mL) substantially suppressed glycolysis (Fig. 4E and F). Because sustained aerobic glycolysis in microglia promotes lactate accumulation and exacerbates tissue injury, lactate production was directly measured. Consistent with this data, VK treatment markedly attenuated OGD/R-induced lactate release (Fig. 4G), further supporting its inhibitory effect on the glycolytic process.

Compared with the control group, OGD/R increased expression of key glycolytic enzymes (*Pfk1*, *Pkm2*, *Pfkfb3)*, which treatment with VK (2, 4, and 8 μg/mL) attenuated these OGD/R-induced effects (Fig. 4H). Meanwhile, OGD/R modulated the expression of TCA cycle-related genes, with downregulation of *Idh1*, *Idh2*, and *Ogdh*, indicating a metabolic reprogramming away from oxidative phosphorylation toward enhanced glycolysis. VK (2, 4, and 8 μg/mL) treatment partially restored the expression of these TCA cycle genes (Fig. 4H), suggesting a shift toward oxidative metabolism.

These data indicate that VK rebalances microglial energy metabolism by enhancing mitochondrial OXPHOS and suppressing excessive glycolysis.

### 3.5 VK restores glucose-mitochondrial carbon flux coupling in primary microglia after OGD/R

**Fig. 5:**
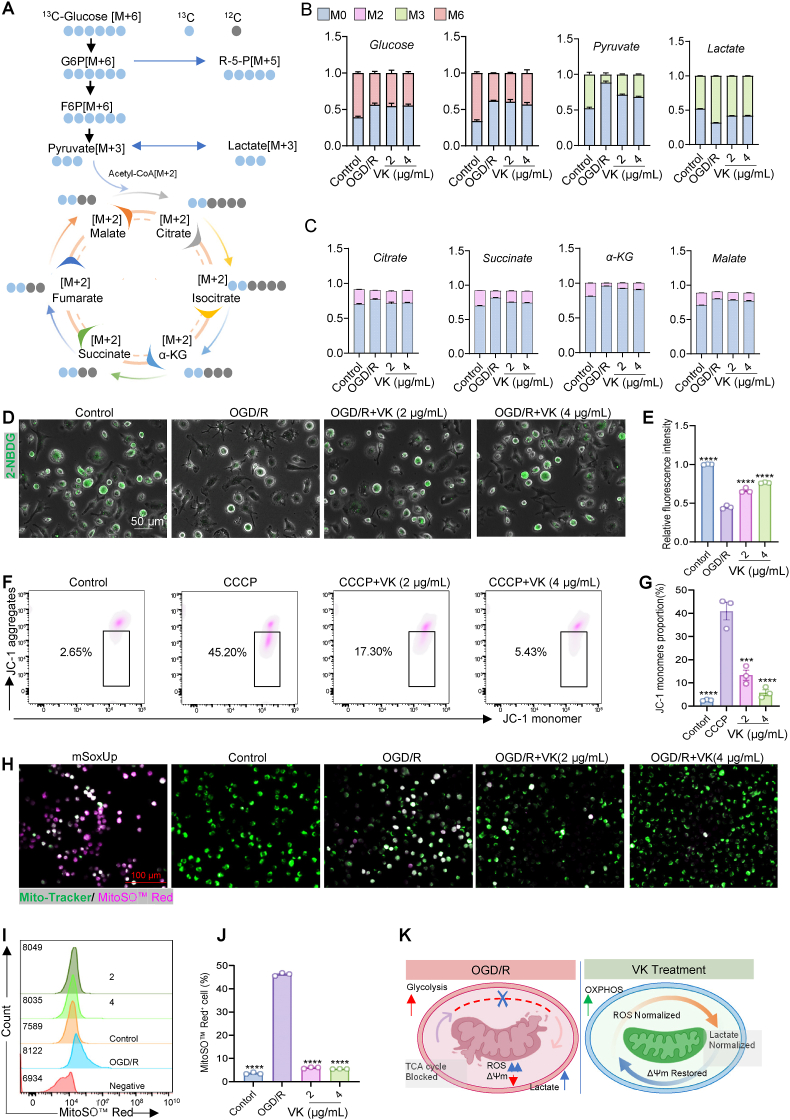
VK restores glucose-mitochondrial carbon flux coupling in microglia following oxygen–glucose deprivation/reoxygenation (OGD/R). (A) Schematic illustration of ^13^C metabolic flux analysis using D-[U–^13^C_6_] glucose tracing, with metabolite detection by liquid chromatography-tandem mass spectrometry (LC-MS/MS). (B, C) Quantification of ^13^C-labeled metabolites in glycolysis/pentose phosphate pathway (PPP); (B) and tricarboxylic acid (TCA) cycle; (C). M + x indicates the number of ^13^C atoms incorporated into each metabolite; (D) Representative fluorescence images of 2-NBDG (glucose uptake) in primary microglia; (E) Quantification of 2-NBDG fluorescence intensity from (D); (F) Representative flow cytometry images of JC-1 staining in primary microglia. JC-1 exists as aggregates in mitochondria with normal membrane potential and as monomers upon depolarization. The shift from red to green indicates loss of mitochondrial membrane potential. CCCP was used as a positive control for depolarization. (G) Quantification of the percentage of JC-1 monomer-positive cells (depolarized mitochondria) from the flow cytometry data shown in (F). (H) Co-localization of MitoSOX™ Red signal (red) with a mitochondrial marker (green) in primary microglia, indicates production of mitochondrial reactive oxygen species (mitoROS); (I) Flow cytometry histograms of MitoSOX™ Red fluorescence; (J) Quantification of MitoSOX™ Red-positive cells; (K) Proposed model illustrating the metabolic dysregulation induced by OGD/R and its restoration by VK. OGD/R stress induces a metabolic decoupling, characterized by enhanced but inefficient glycolysis (as shown by lactate accumulation and diminished TCA cycle labeling), mitochondrial dysfunction (loss of ΔΨm), and oxidative stress (MitoSOX™ Red). VK intervention restores metabolic flux into the TCA cycle, thereby rescuing mitochondrial bioenergetics and reducing ROS production. Data are presented as mean ± SEM (n = 3 independent experiments); one-way ANOVA with Tukey's post hoc test: ∗*P* < 0.05, ∗∗*P* < 0.01, ∗∗∗*P* < 0.001, ∗∗∗∗*P* < 0.0001 vs OGD/R group.

To mechanistically define how VK reprograms microglial metabolism, we employed ^13^C isotopic flux analysis using uniformly labeled glucose (Fig. 5A) [45,46]. OGD/R caused markedly dysregulation of central carbon metabolism, characterized by a significant reduction in intracellular ^13^C_6_-glucose (M+6) and ^13^C_6_-fructose-6-phosphate (F6P, M+6), indicating accelerated glycolytic consumption. Concomitantly, ^13^C_3_-labeled lactate (M+3) accumulated, whereas ^13^C_3_-labeled pyruvate (M+3) was decreased (Fig. 5B). Consistent with these flux changes, LDH activity and its protein expression was markedly elevated following OGD/R (see Supplementary Methods 1.5), supporting enhanced conversion of pyruvate to lactate (Supplementary Fig. 7). Critically, labeling of key TCA cycle intermediates—including ^13^C_2_-citrate (M+2), ^13^C_2_-α-ketoglutarate (M+2), ^13^C_2_-succinate (M+2), and ^13^C_2_-malate (M+2)—was markedly reduced, confirming severe disruption of mitochondrial oxidative metabolism and a decoupling of glycolysis from the TCA cycle (Fig. 5C). Notably, VK treatment effectively reversed these metabolic abnormalities, demonstrating its capacity to restore glucose-mitochondrial carbon flux coupling.

Supporting this conclusion, 2-NBDG fluorescence assays revealed that OGD/R induced impaired glucose uptake, which was normalized by VK treatment (Fig. 5D and E). Furthermore, VK preserved mitochondrial membrane potential (MMP). Compared with the control group, CCCP, a mitochondrial oxidative phosphorylation uncoupler, induced severe mitochondrial depolarization, as evidenced by a marked increase in JC-1 monomers. VK pretreatment reversed this effect, restoring mitochondrial membrane potential integrity (Fig. 5F and G). Fluorescence microscopy images further validated these findings, showing consistent restoration of JC-1 aggregates by VK treatment (Supplementary Fig. 8A). The restoration of mitochondrial integrity was functionally linked to a substantial reduction in oxidative stress, as VK markedly attenuated the OGD/R-driven increase in mitochondrial superoxide production, as quantified by fluorescence microscopy and flow cytometry (Fig. 5H–J). Flow cytometric quantification of ROS-positive cells further corroborated these findings (Supplementary Fig. 8B and 8C; as described in Supplementary Methods 1.6).

Taken together, these data delineate a coherent mechanism by which VK restores metabolic homeostasis after ischemic insult: it reduces glycolytic flux, enhances pyruvate entry into mitochondria, replenishes the TCA cycle, and thereby restores efficient oxidative phosphorylation while quenching associated oxidative stress (Fig. 5K).

### 3.6 VK reconstructs the arginine**-**TCA cycle axis to drive metabolic reprogramming

**Fig. 6:**
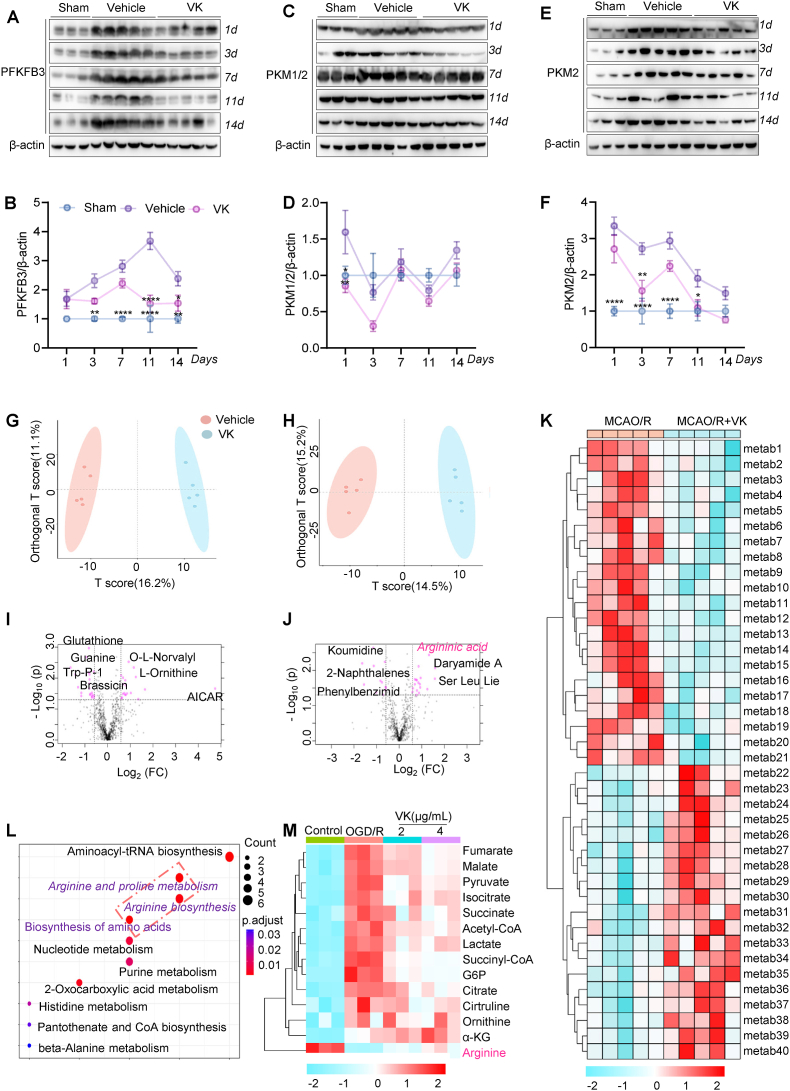
VK reconstructs the arginine-TCA cycle axis to drive metabolic reprogramming. (A-F) Representative Western blots and corresponding quantification of glycolytic enzymes—6-phosphofructo-2-kinase/fructose-2,6-bisphosphatase 3, pyruvate kinase isoform M1/2, and pyruvate kinase isoform M2—in the ischemic hemisphere of MCAO/R vehicle and VK-treated mice at indicated time points. Data were normalized to the sham-operated group. Data are presented as mean ± SEM (n = 3–5 mice/group); two-way ANOVA followed by Bonferroni's post hoc test: ∗*P* < 0.05, ∗∗*P* < 0.01,∗∗∗*P* < 0.0001 vs Vehicle at the same time point; (G, H) Principal component analysis (PCA) score plots of brain metabolomes in positive (G) and negative (H) ion modes; (I, J) Volcano plots of differentially abundant metabolites inpositive (I) and negative (J) ion modes. Significance threshold: |log_2_ (fold change) |>0.5 and adjusted *P* < 0.05; (K) Hierarchical clustering heatmap of significantly altered metabolites, highlighting VK-induced accumulation of arginine-related intermediates; (L) Kyoto Encyclopedia of Genes and Genomes (KEGG) pathway enrichment analysis (dot plot) revealing VK-specific activation of arginine and proline metabolism pathways; (M) Heatmap showing metabolite changes in OGD/R-challenged primary microglia with or without VK treatment (n = 3 independent experiments).

To investigate the metabolic basis of VK's effects, we first evaluated the dynamic expression of key glycolytic enzymes in the ischemic hemisphere. PFKFB3, a key glycolytic activator, was markedly upregulated in the vehicle group from days 3–11 post-MCAO/R, reaching its peak on day 11. VK treatment persistently suppressed this elevation during days 3–7 and further reduced its expression on days 11–14 (Fig. 6A and B). PKM2 protein expression was significantly increased from 1 to 11 days after surgery and gradually decreased until day 14, suggesting that MCAO/R injury markedly activates the glycolytic key isoform PKM2. Compared with the vehicle group, VK treatment overall downregulated PKM1/2 expression, with the most significant inhibitory effect at 1 day after surgery, and also significantly reduced PKM2 expression, especially at 3 and 11 days, thereby effectively blocking MCAO/R-induced excessive activation of glycolysis (Fig. 6C–F).

Untargeted metabolomics revealed that VK substantially reshaped the post-ischemic cerebral metabolome (Fig. 6G and H), with clear separation from the MCAO/R group in PCA. Among 1664 identified metabolites, 71 were significantly altered (38 upregulated and 33 downregulated) in the VK group compared with the MCAO/R group (defined as |log_2_ (fold change) |>0.5 and adjusted *P* < 0.05) (Fig. 6I and J). Hierarchical clustering highlighted a distinct metabolic signature: MCAO/R mice accumulated markers of L-aspartyl-4-phosphate (an ATP synthesis inhibitor), oxidized glutathione (an indicator of oxidative stress), and Trp-P-1 (a neurotoxin), suggesting energy depletion and oxidative injury. Conversely, the VK group exhibited elevated levels of arginine-related intermediates, including arginine, xanthine, and citicoline (Fig. 6K; Table 2-described metabolites), implicating arginine-centered mitochondrial energy pathways in restoring post-ischemic metabolic homeostasis. Pathway enrichment specifically identified “Arginine and proline metabolism” and “Arginine biosynthesis” as top pathways activated by VK (Fig. 6L).

**Table 2:** Metabolite annotation.

Number	Metabolite	Biological Function
metab1	Callicarpenal	Antibacterial
metab2	Orcinol glucoside	Antioxidant
metab3	Dihydro-2-methylfuranthione	Antioxidant
metab4	L-Aspartyl-4-phosphate	Intermediate of amino acid metabolism
metab5	lsorhamnetin-3-0-glucoside	Antioxidant
metab6	Serotonin	Neurotransmitter
metab7	Koumidine	Immunoregulation
metab8	Glutathione disulfide	Antioxidant and detoxification
metab9	2-Naphthalenesulfonic acid	exogenous metabolic detoxification
metab10	8-Methylthiooctanaldoxime	Sulfur metabolism intermediate
metab11	Senedigitalene	Purgative
metab12	Brassicin	Anticancer
metab13	Acerosin	Hepatoprotection
metab14	Octylamine	Vascular regulation
metab15	Citicoline	Neuroprotection
metab16	Guanosine	Precursor of nucleoside synthesis
metab17	(R)-2-Aminoheptanoic acid	Antibacterial
metab18	Guanine	Nucleic acid constituent
metab19	UDP-glucuronic acid	Glycosylation donor
metab20	Tanaparthin alpha-peroxide	Antifungal
metab21	Trp-P-1	Mutagenic
metab22	5-Methyluridine	RNA epigenetic regulation
metab23	N-Stearoyltaurine	Lipid metabolism regulation
metab24	Indole-3-carbinol	Anticancer
metab25	l-Histidine	Protein amino acid synthesis
metab26	Brunfelsamidine	Metabolic substrate of alkaloids
metab27	Gamma-Glu-Gly	Precursor of glutathione synthesis
metab28	O-L-Norvalyl-5-isourea	Metabolic regulation
metab29	Argininic acid	Intermediate of urea cycle
metab30	Propanoic acid	Thiol modification
metab31	H-ILE-TRP-OH	Immunoregulation
metab32	Daryamide A	Antibacterial
metab33	Prolyl-Gamma-glutamate	Cellular signal regulation
metab34	Hexanal-1,3-octanediol acetal	Oxidative stress
metab35	Arachidic Acid	Biomembrane component
metab36	N-Carboxymethionine	Antioxidant
metab37	Ser Leu lle	Cell proliferation regulation
metab38	Xanthine	Intermediate of purine metabolism
metab39	N-Lactoyl-Phenylalanine	Metabolic reprogramming
metab40	1-Carboxyethyltyrosine	Oxidative stress

Mechanistically, VK modulated the arginine metabolic axis following CIRI. Both OGD/R in primary microglia and MCAO/R in mouse brain tissue significantly induced NO elevation. VK treatment suppressed this abnormal NO increase (Supplementary Fig. 9; as described in Supplementary Methods 1.7), indicating that VK attenuates the dysregulated arginine-NO metabolism induced by CIRI. This restoration was critical because ASL cleaves argininosuccinate to generate arginine and fumarate—a key TCA cycle intermediate—thereby directly coupling arginine metabolism to mitochondrial energy production [47]. We next asked whether these metabolic alterations occur specifically in microglia. Immunofluorescence co-localization analysis in brain tissue showed that VK restored Glut1 expression and normalized elevated PFKFB3 levels at the microglia-neuron interface (IBA1^+^NeuN^+^ regions; Supplementary Fig. 10).

To validate these findings, we conducted untargeted metabolomic profiling of primary microglia subjected to OGD/R with or without VK treatment, as described in Supplementary Methods 1.8 (Fig. 6M). OGD/R exposure led to marked accumulation of multiple TCA cycle intermediates, including citrate, succinate, fumarate, and malate, as well as increased levels of lactate and pyruvate, suggesting TCA cycle stalling rather than enhanced oxidative metabolism (Fig. 6M). Notably, arginine levels were decreased after OGD/R and partially restored by VK treatment. Consistent with impaired glucose uptake, glucose-6-phosphate (G6P) was decreased after OGD/R, and VK treatment slightly, though not significantly, restored its level. Together, these findings demonstrate that VK remodels central carbon metabolism in microglia by restoring TCA cycle flux and reversing the metabolic stalling induced by OGD/R.

### 3.7 VK sustains OXPHOS by stimulating microglial arginine metabolism

**Fig. 7:**
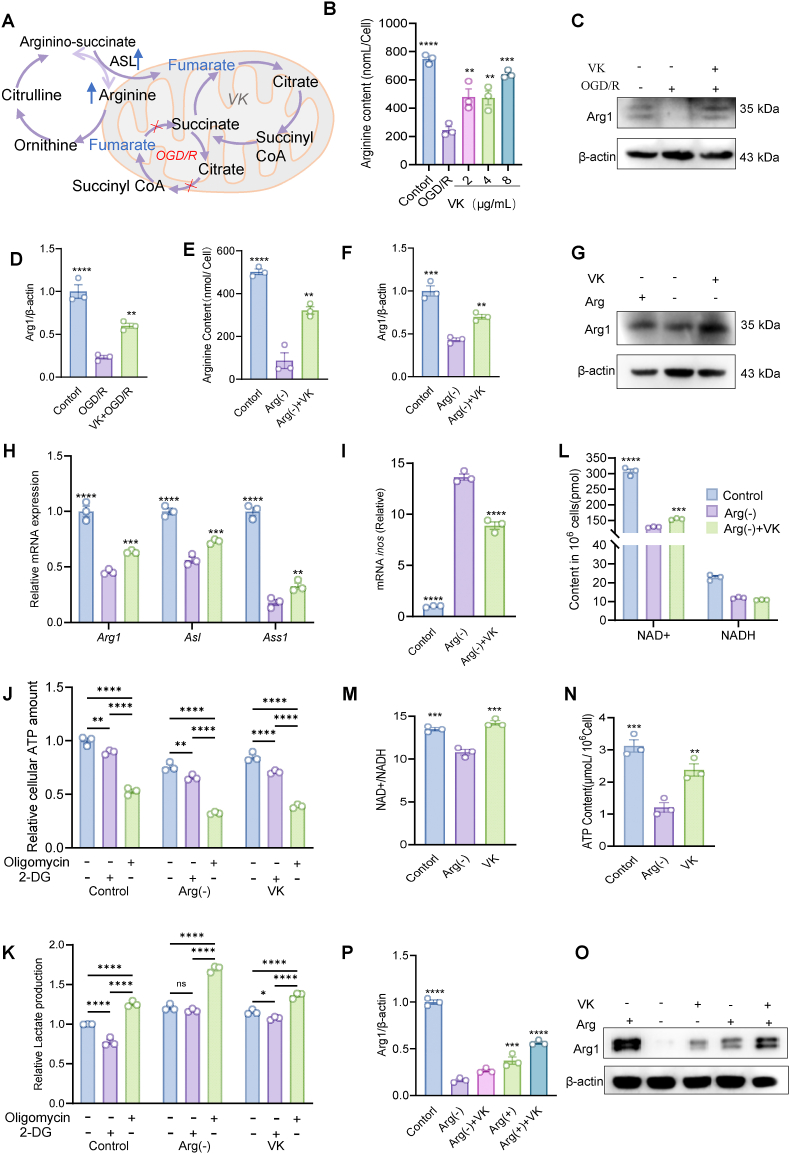
VK promotes microglial oxidative phosphorylation under metabolic stress by enhancing arginine metabolism. (A) Schematic illustrating VK-mediated restoration of TCA cycle flux via the arginine-fumarate axis. OGD/R impairs argininosuccinate lyase (ASL) activity, leading to TCA cycle stalling and accumulation of fumarate and succinate. VK enhances ASL-catalyzed conversion of argininosuccinate (ASA) to arginine and fumarate, restoring TCA cycle flux and resolving metabolite accumulation. (B) Intracellular arginine levels in OGD/R-insulted primary microglia treated with VK (2, 4, and 8 μg/mL), measured by arginine assay kit. (C, D) Representative Western blots and quantification of arginase-1 (Arg1) protein expression in OGD/R-exposed microglia. (E) Intracellular arginine concentrations in microglia cultured in arginine-depleted medium, with or without VK supplementation (4 μg/mL). (F, G) Representative Western blots and quantification of Arg1 in arginine-depleted microglia. (H, I) Relative mRNA expression of arginine metabolic enzymes in arginine-depleted microglia, measured by qRT-PCR. VK upregulated *asl*, *ass1*, and arg*1* (H) while downregulating *inos* (I). (J, K) Metabolic flexibility evaluated by ATP production and lactate release following sequential inhibition of OXPHOS (oligomycin) and glycolysis (2-DG) in arginine-depleted microglia, with or without VK supplementation. (L, M) Cellular NAD^+^ and NADH levels (L) and NAD^+^/NADH ratio (M) in arginine-depleted microglia. (N) Intracellular ATP levels measured by ATP assay kit. (O, P) Representative Western blots and quantification of Arg1 in microglia with arginine replenishment (±VK pretreatment). Data are mean ± SEM (n = 3 independent experiments); one-way ANOVA with Tukey's post hoc test: ∗*P* < 0.05, ∗∗*P* < 0.01, ∗∗∗*P* < 0.001.

To test whether VK modulates microglial metabolism through arginine regulation, we employed an *in vitro* OGD/R model. As illustrated in the mechanistic model (Fig. 7A), we hypothesized that VK restores TCA cycle flux by enhancing the arginine-fumarate axis: OGD/R impairs ASL activity, leading to TCA cycle stalling and accumulation of fumarate and succinate (Fig. 6L), while VK enhances ASL-catalyzed conversion of argininosuccinate to arginine and fumarate, thereby relieving the metabolic bottleneck and restoring TCA cycle flux (Fig. 6M).

Consistent with this model, OGD/R significantly depleted intracellular arginine, whereas VK treatment restored arginine levels in a dose-dependent manner, with the most pronounced effect observed at 8 μg/mL (Fig. 7B). Immunoblotting analysis confirmed that VK upregulated Arg1, a key enzyme that channels arginine toward polyamine and proline synthesis (Fig. 7C and D). To determine whether VK can stimulate arginine metabolism independently of exogenous arginine, we next cultured primary microglia in arginine-deficient medium, as described in Supplementary Methods 1.9. Under these conditions, VK treatment still enhanced *de novo* arginine biosynthesis (Fig. 7E) and increased Arg1 protein expression (Fig. 7F and G), indicating that VK activates the intrinsic arginine metabolic pathway even when extracellular arginine is limited. This restoration of arginine metabolism was functionally linked to TCA cycle recovery, as VK upregulated the expression of *Asl*, *Ass1*, and Arg*1* mRNA (Fig. 7H) while downregulating *Inos* mRNA (Fig. 7I)—consistent with enhanced ASL activity and fumarate generation, as well as a shift away from the pro-inflammatory iNOS branch—and resolved the OGD/R-induced accumulation of fumarate and succinate (Fig. 6M).

We next assessed the functional consequences of arginine depletion on cellular energy metabolism. Arginine deprivation compromised OXPHOS-dependent ATP production. To evaluate metabolic flexibility, we employed pathway-specific inhibitors: 2-DG (a glycolysis inhibitor) and oligomycin (an OXPHOS inhibitor). In all groups, 2-DG treatment reduced ATP production and lactate levels, confirming the contribution of glycolysis to cellular energy metabolism. Oligomycin treatment decreased ATP to very low levels across all groups while inducing a compensatory increase in lactate production. Notably, the arginine-deprived group exhibited reduced basal ATP levels and a more pronounced compensatory glycolytic response to oligomycin, as indicated by elevated lactate production. VK treatment largely preserved ATP levels and attenuated the excessive lactate production induced by oligomycin (Fig. 7J and K), suggesting that VK ameliorates the energy imbalance caused by arginine deficiency.

Additionally, arginine deprivation also depleted NAD^+^ levels and reduced the NAD^+^/NADH ratio; both were partially restored by VK treatment (Fig. 7L and M). Direct measurements confirmed that VK intervention significantly elevated intracellular ATP levels that were compromised by arginine deprivation (Fig. 7N). Finally, arginine supplementation increased Arg1 protein expression in microglia, and co-treatment with VK further augmented Arg1 levels, indicating a synergistic effect of VK with exogenous arginine (Fig. 7O and P).

Cumulatively, these findings demonstrate that VK restores energy metabolism and redox homeostasis by stimulating the intrinsic arginine metabolic pathway, thereby preserving OXPHOS and supporting microglial function after metabolic stress.

### 3.8 VK coordinates microglial metabolism and inflammation via the AMPK/mTOR signaling axis

**Fig. 8:**
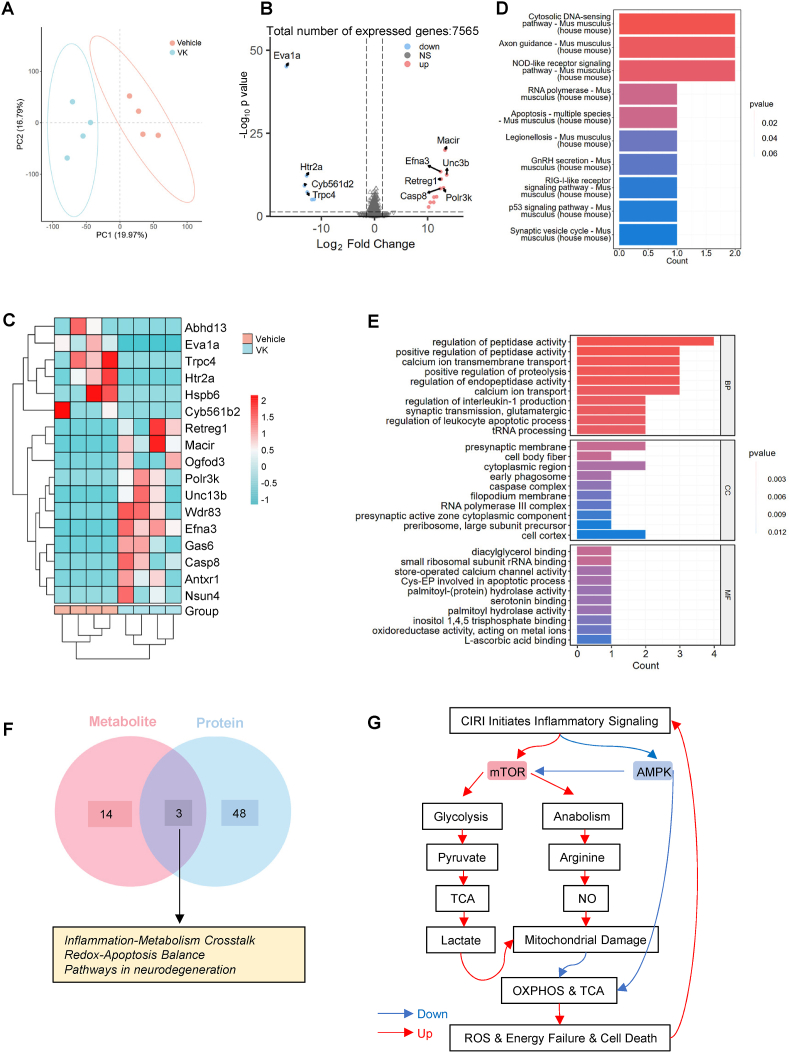
Multi-omics integration identifies the AMPK/mTOR axis as a key mediator of VK-induced metabolic and inflammatory regulation. (A) PCA score plot of proteomic profiles showing clear separation between middle cerebral artery occlusion/reperfusion (MCAO/R) and VK-treated groups; (B) Volcano plot of differentially expressed proteins (DEPs) between MCAO/R and VK-treated groups in the ischemic hemisphere |log_2_ (fold change) |>1.5, adjusted *P* < 0.05; (C) Hierarchical clustering heatmap of DEPs across experimental conditions; (D) KEGG pathway enrichment analysis of DEPs (e.g., cytosolic DNA-sensing pathway, axon guidance); (E) GO enrichment analysis of DEPs categorized by biological process (BP), cellular component (CC), and molecular function (MF); (F) Integrative network linking metabolomic and proteomic modules (arginine metabolism, glutathione metabolism, neurotransmitter metabolism), highlighting crosstalk between inflammation, oxidative stress, and neuronal signaling; (G) Proposed mechanistic model: MCAO/R disrupts the AMPK/mTOR axis, driving microglial metabolic dysfunction and neuroinflammation. VK restores this balance, shifting metabolism toward OXPHOS and suppressing inflammation to promote neuroprotection.

To elucidate the upstream signalling mechanisms through which VK orchestrates microglial reprogramming, we conducted a quantitative proteomic analysis of the ischemic hemisphere. PCA revealed a clear separation between the MCAO/R and the VK-treated groups (Fig. 8A). Differential expression analysis identified 17 significantly altered proteins—11 upregulated and 6 downregulated (|log_2_ (fold change) |>1.5, adjusted *P* < 0.05) in the VK-treated mice. Notably, VK treatment markedly upregulated MACIR (a protein associated with neuronal survival and inflammatory regulation) while downregulating Eva1a (an Eph receptor-interacting protein linked to apoptosis and synaptic dysfunction) (Fig. 8B and C). This bidirectional modulation suggests that VK concurrently enhances endogenous neuroprotective pathways and suppresses detrimental processes.

KEGG analysis of the proteomic data indicated that VK significantly influenced several signalling pathways, including the cytosolic DNA-sensing pathway (involved in inflammation and cell death regulation), axon guidance (promoting neural regeneration), and NOD-like receptor signalling (modulating inflammasome activation) (Fig. 8D). GO analysis further supported VK's broad biological impact, with enrichment in processes such as “peptidase regulation” (potentially preserving BBB integrity) and cellular components including the “presynaptic membrane” (suggesting synaptic protection) (Fig. 8E). These findings illustrate VK's integrated potential to modulate inflammation, promote neuronal remodelling, and regulate enzymatic activity.

Integration of metabolomic and proteomic datasets revealed three core functional modules significantly modulated by VK: arginine metabolism, glutathione metabolism, and neurotransmitter metabolism (Fig. 8F). These modules intersected with proteomic pathways governing inflammation (e.g., NOD-like receptor and TNF signalling), oxidative stress (e.g., apoptosis and necroptosis), and neuroactive ligand-receptor interactions, forming a cohesive network that underscores the interplay among inflammation-metabolism crosstalk, redox-apoptosis balance, and neurodegeneration pathways (Fig. 8F). For example, alterations in arginine metabolites (e.g., increased l-ornithine) and apoptosis-related proteins (e.g., increased Casp8) suggested a rebalancing of the arginine-NO axis, reducing its potential to promote inflammation and cell death.

Based on these multi-omics findings, we propose a mechanistic model in which CIRI induces a core metabolic disturbance characterized by hyperactivation of the mTOR pathway and suppression of AMPK (Fig. 8G). This aberrant anabolic state promotes glycolysis and protein synthesis while impairing mitochondrial OXPHOS, leading to lactate overproduction and acidosis. Concurrently, excessive anabolic and inflammatory demands redirect arginine metabolism toward excessive NO generation, producing cytotoxic peroxynitrite that exacerbates mitochondrial damage. The resulting energy supply-demand mismatch triggers a vicious cycle of ROS overproduction and cell death. VK is proposed to interrupt this cycle by rebalancing the AMPK/mTOR axis, thereby restoring metabolic homeostasis and mitigating inflammatory response.

### 3.9 Restoration of glycolytic metabolism and inflammatory homeostasis by VK requires the AMPK/mTOR pathway

**Fig. 9:**
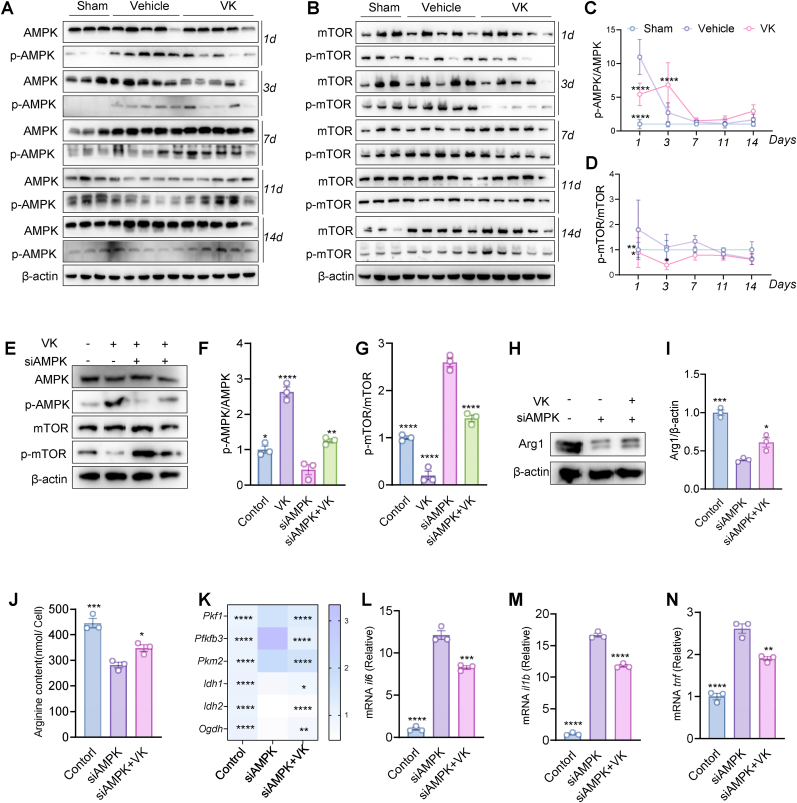
VK restores metabolic and inflammatory homeostasis in an AMPK/mTOR-dependent manner. (A, C) Representative Western blots (A) and quantification (C) of AMPK and phosphorylated AMPK (p-AMPK, Thr172) in the ischemic hemisphere at indicated time points post-MCAO/R; (B, D) Representative Western blots (B) and quantification (D) of mTOR and phosphorylated mTOR (p-mTOR, Ser2448). Data were normalized to the sham-operated group. Data are mean ± SEM (n = 3−5 mice per group); two-way ANOVA with Bonferroni's post hoc test: ∗*P* < 0.05, ∗∗*P* < 0.01 vs MCAO/R vehicle group; (E–G) Representative Western blots (E) and quantification (F, G) of AMPK, p-AMPK, mTOR, and p-mTOR in OGD/R-challenged microglia transfected with AMPK-targeting siRNA (siAMPKα) or non-targeting control siRNA; (H, I) Representative Western blots (H) and quantification (I) of Arg1 in AMPK-silenced microglia; (J–N) Functional consequences of AMPK knockdown: intracellular arginine levels (J), mRNA expression of OXPHOS-related (*Idh1*, *Idh2*, *Ogdh*) and glycolytic (*Pkf1*, *Pkm2*, *Pfkfb3*) genes (K), and mRNA levels of pro-inflammatory cytokines (*Il6*, *Il1b*, *Tnf*) (L–N). Data are mean ± SEM (n = 3 independent experiments); one-way ANOVA with Tukey's post hoc test: ∗*P* < 0.05, ∗∗*P* < 0.01, ∗∗∗*P* < 0.001,∗∗∗*P* < 0.0001 vs siAMPKα groups.

To define the temporal dynamics of key energy-sensing pathways, we monitored AMPK and mTOR activation after MCAO/R. The p-AMPK/AMPK ratio sharply increased on day 1 and subsequently normalized (Fig. 9A and C). VK treatment modulated this response, resulting in a lower p-AMPK/AMPK ratio than the MCAO/R group on day 1 but sustaining higher activation levels on day 3 (Fig. 9A and C). This biphasic regulation suggests that VK fine-tunes AMPK, a master energy sensor that, under early energy stress, inhibits the anabolic driver mTORC1—primarily through phosphorylation of TSC2 and Raptor [48]—thereby promoting catabolic processes such as autophagy during energy-deficient states. Correspondingly, the p-mTOR/mTOR ratio was initially elevated on day 1 post-MCAO/R but progressively declined to sub-baseline levels by days 11−14. VK intervention robustly suppressed mTOR activity throughout this period (Fig. 9B and D).

Given the established crosstalk between mTORC1 activity and arginine availability [49], and supported by our metabolomic data showing VK-induced accumulation of arginine-pathway intermediates, these findings indicate that VK coordinates arginine metabolism through the AMPK/mTOR signalling axis. Specifically, VK attenuates early AMPK hyperactivation—mitigating acute energy stress—while sustaining AMPK activity and prolonging mTOR suppression in later phases, thereby promoting arginine recycling and facilitating long-term metabolic recovery.

To establish a causal role for AMPK, we performed siRNA-mediated AMPK knockdown. Silencing AMPK effectively reversed VK-induced alterations in p-AMPK and p-mTOR levels (Fig. 9E–G). Functionally, AMPK inhibition abolished the metabolic benefits of VK, as Arg1 expression reverted to OGD/R stress (Fig. 9H and I). Moreover, AMPK knockdown blocked the anti-inflammatory and M1-to-M2-polarizing effects of VK (Fig. 9J–N). Bio-Layer Interferometry (BLI) confirmed a direct, moderate-affinity interaction between VK and AMPK, with a dissociation constant (KD) of 3.901 × 10^−7^ M (Supplementary Fig. 11; Supplementary Methods 1.10 for detailed procedures). Collectively, these results demonstrate that VK restores metabolic balance and suppresses neuroinflammation in an AMPK/mTOR-dependent manner.

## 4 Discussion

Our study delineates an immunometabolic axis through which the venom-derived peptide VK attenuates neuroinflammation and oxidative stress after CIRI in mice and in primary microglia. We demonstrate that VK's neuroprotection is orchestrated by a fundamental reprogramming of microglial metabolism, shifting the balance from aerobic glycolysis toward OXPHOS and concurrently promoting a transition from the pro-inflammatory M1 to a reparative M2 phenotype. Crucially, we also provide evidence that this reprogramming is driven by VK-mediated AMPK activation and mTOR inhibition, which in turn reconstructs the arginine-TCA cycle metabolic axis to restore mitochondrial bioenergetics and redox homeostasis (Fig. Graphical abstract).

This work places VK within the growing field of immunometabolism in neurological diseases [50]. Seminal studies [51] have established that microglia, like peripheral macrophages, adopt glycolytic metabolism during pro-inflammatory activation—a phenomenon analogous to the Warburg effect. However, therapeutic agents capable of reversing the pathological glycolytic shift in stroke remain scarce. We not only confirm this metabolic switch in the context of CIRI but also demonstrate that VK acts as a potent pharmacological agent capable of reversing it. While HIF-1α and NF-κB are recognized as downstream mediators of glycolytic activation in inflammation [52], our data reveal that VK operates upstream of the energy-sensing AMPK/mTOR hub [53] to redirect the entire metabolic program. This upstream targeting offers a strategic advantage by simultaneously modulating multiple downstream effector pathways.

A key innovation of this study is the identification of arginine metabolism as a critical effector downstream of VK-induced AMPK/mTOR signalling. Although arginine metabolism is a well-established determinant of macrophage polarization in peripheral tissues [54], its role in microglial metabolic adaptation following CIRI has remained poorly characterized and often oversimplified. Our integrated omics approach shows that VK not only elevates intracellular arginine levels but also enhances its channelling into the TCA cycle via fumarate generation—a direct molecular link between arginine catabolism and mitochondrial energetics [55]. This mechanistic insight explains the sustained OXPHOS observed in VK-treated cells. Nevertheless, we note that the evidence for arginine carbon directly entering the TCA cycle via fumarate is indirect (based on arginine levels, ASL mRNA, and steady-state fumarate). Direct isotopic tracing with U–^13^C-arginine has not been performed, which is a limitation of the current study.

Notably, this arginine-TCA cycle coupling is conserved but exhibits cell-type and species-specific differences: in humans, Arg1 is predominantly expressed in neutrophils (stored in tertiary granules), whereas in mice, it is mainly localized in macrophages (cytosol) [56]. This species divergence highlights the importance of validating VK's effects in human-derived systems (e.g., iPSC-derived microglia) for clinical translation, as rodent models may not fully recapitulate human arginine metabolic regulation. Additionally, arginine metabolites exert dual roles in ischemic injury: while l-ornithine can suppress CD8^+^ T cell function and promote tumor progression, it can also detoxify ammonia in ammonia-accumulating microenvironments (e.g., post-ischemic brain) to enhance T cell activity [57]. Our data show that VK increases arginine and citrulline levels while reducing excessive ornithine accumulation, suggesting it finely tunes arginine metabolic branching to favor reparative outcomes—an effect consistent with the “metabolic rescue” role of citrulline-arginine recycling in T cells [58]. Our findings, therefore, advance the field beyond correlative observations—such as the association between arginine deprivation and exacerbated ischemic injury [59,60]—by identifying a specific therapeutic agent that actively restores this pathway and delineates its upstream regulatory control [61].

Our integrated metabolic flux analysis provides direct evidence for the glycolytic shift in activated microglia and its reversal by VK. Following OGD/R, we observed upregulation of PKM2 and increased LDH activity, which collectively enhance glycolytic flux. ^13^C-glucose tracing confirmed this shift: lactate M3 was elevated while pyruvate M3 was reduced. This apparent divergence is consistent with the “leaky cell” model described by Quek et al. [62], where fast exchange fluxes [63] around the pyruvate node cause dilution of intracellular pyruvate ^13^C enrichment by accumulated extracellular lactate—a scenario exacerbated by OGD/R-induced LDH upregulation. VK treatment reversed this pattern, downregulating PKM2 and LDH, and restoring lactate M3 and pyruvate M3 to near-control levels. This metabolic redirection aligns with VK-induced AMPK activation and mTOR inhibition, supporting its neuroprotective mechanism.

At the molecular level, we delineate a coherent signalling–metabolism cascade. VK activates AMPK, thereby inhibiting mTORC1. This suppression of anabolic mTOR signalling downregulates glycolytic enzyme expression (e.g., *pkm2*, *pfkfb3*) while upregulating genes involved in OXPHOS (e.g., *idh1*, *ogdh*). Concurrently, the VK-induced increase in intracellular arginine levels supports Arg1-mediated flux, which serves a dual purpose: it promotes the immunomodulatory programs of M2 polarization and, through ASL-catalyzed fumarate production, directly replenishes the TCA cycle to sustain mitochondrial ATP synthesis. This crosstalk represents a sophisticated regulatory network that enables microglia to meet the dynamic bioenergetics demands of the injured brain by coupling energy sensing, metabolic reprogramming, and immune function.

Our study also highlights the temporal dynamics of metabolism-neuroinflammation coupling during CIRI. The early, transient activation of AMPK post-injury likely reflects a physiological compensatory response to acute energy stress, whereas its subsequent decline may contribute to the failure of inflammation resolution [17]. VK appears to fine-tune this temporal pattern by amplifying early AMPK activation to prevent energy crisis-induced damage and by reinforcing its activity in later phases to promote a sustained metabolic and functional shift toward repair. This phase-specific regulation underscores the potential superiority of VK over conventional AMPK agonists and emphasizes the importance of timing in targeting immunometabolic pathways therapeutically. Additionally, the upregulation of methylated arginines (ADMA, SDMA) in dementia [64] and cardiovascular disease [65] suggests that arginine metabolic dysregulation is a common driver of neuroinflammation across neurological disorders—further supporting VK's broad translational potential beyond stroke.

Several limitations of this study warrant consideration, as they inform directions for future research. First, as noted above, we have not performed direct isotopic tracing with U-^13^C arginine to definitively prove that arginine-derived carbon enters the TCA cycle via fumarate. Future studies using such tracing are needed. Second, although we have established the central role of the AMPK/mTOR signalling axis, the specific receptor through which VK initiates this cascade remains unknown. Future studies employing approaches such as photoaffinity labeling or CRISPR-based functional screening could help identify this upstream molecular target. Third, our *in vivo* experiments primarily evaluated short-term outcomes; long-term survival studies are needed to determine whether VK-induced metabolic reprogramming translates into durable cognitive and motor recovery and confers lasting neuroprotection. Moreover, VK administration was initiated 1 h after reperfusion; whether delayed dosing (e.g., 6 h or 24 h) retains similar neuroprotective and metabolic effects remains unknown and should be addressed in future studies. Fourth, the translational relevance of our findings requires validation in human-based systems, such as iPSC-derived microglia or post-mortem human tissue exposed to ischemic conditions—especially given the species-specific differences in arginase expression and arginine metabolism [52,65]. Fifth, while we focused on microglia, other brain cells (e.g., astrocytes and neurons) also possess active arginine metabolic pathways, and whether VK modulates their metabolic state remains unexplored. Future studies using cell-type-specific knockout models will be necessary to dissect the cell-autonomous versus non-cell-autonomous effects of VK. Finally, although our data support a strong association between arginine metabolic rewiring and M2 polarization, a direct causal link—for instance, rescuing the M2 phenotype by exogenous arginine or fumarate in AMPK-knockdown cells—remains to be formally established.

Despite these limitations, our findings have broad implications. This work establishes the AMPK/mTOR-arginine metabolic axis as a central, targetable pathway in post-ischemic neuroinflammation. Therapeutically, VK represents a prototype of integrated neuroprotection, simultaneously rescuing metabolic dysfunction and quenching inflammation—a dual strategy with potential advantages over single-target agents. Its mechanism converges with emerging immunometabolic therapies, underscoring its relevance to future drug development. Future efforts should focus on: validating this axis in human-derived models to address species-specific differences; optimizing VK's drug-like properties to enhance CNS delivery; and testing its efficacy in other neuroinflammatory conditions where microglial metabolism is compromised. Ultimately, our findings provide a mechanistic blueprint for treating ischemic brain injury through coordinated metabolic and immunomodulatory reprogramming.

## 5 Conclusions

In summary, this study identifies the wasp venom-derived peptide VK as a novel immunometabolic reprogramming agent that confers neuroprotection after CIRI. The central mechanistic insight is the delineation of the AMPK/mTOR-arginine-TCA cycle coupling axis: VK activates AMPK and inhibits mTOR to enhance arginine metabolism, which is functionally coupled to the TCA cycle via fumarate production. This metabolic rewiring replenishes mitochondrial bioenergetics and restores redox balance, thereby shifting microglia from a glycolysis-dependent pro-inflammatory M1 phenotype toward an OXPHOS-supported pro-repair M2 phenotype.

By directly linking arginine-centered metabolic reprogramming to the suppression of neuroinflammation, VK addresses two intertwined pathological pillars of post-stroke injury. These findings not only elucidate a fundamental mechanism in stroke biology but also nominate VK as a promising therapeutic candidate embodying a multi-target “metabolic immunotherapy” strategy. This work establishes a mechanistic blueprint for developing natural product-derived agents that modulate immunometabolic crosstalk. Future studies should focus on validating this axis in human-relevant models and optimizing CNS-targeted delivery of VK to advance its clinical translation.

## CRediT authorship contribution statement

**Dexiao Wang:** Data curation, Formal analysis, Methodology, Visualization, Writing – original draft. **Jingyu Zhang:** Data curation, Formal analysis, Methodology, Writing – original draft. **Zhejun Zhuang:** Data curation, Methodology, Visualization, Writing – original draft. **Qian Wang:** Methodology. **Jie Li:** Methodology. **Xue Wang:** Methodology. **Kunkun Li:** Methodology. **Yunyun Liu:** Methodology. **Yanhui Cao:** Methodology. **Lijuan Li:** Resources, Supervision. **Yunwu Zhang:** Conceptualization, Investigation. **Yu Zhao:** Conceptualization, Investigation, Resources. **Yingjun Zhao:** Conceptualization, Funding acquisition, Project administration, Supervision, Writing – review & editing. **Hairong Zhao:** Conceptualization, Funding acquisition, Methodology, Project administration, Resources, Supervision, Writing – original draft, Writing – review & editing. **Chenggui Zhang:** Conceptualization, Funding acquisition, Project administration, Software, Supervision, Writing – original draft, Writing – review & editing.

## Declaration of competing interest

The authors declare that they have no known competing financial interests or personal relationships that could have appeared to influence the work reported in this paper.

## Data Availability

The data will be made available upon reasonable request. Raw flow cytometry data (.fcs files) are available in figshare (https://doi.org/10.6084/m9.figshare.32171751). The raw data underlying the main figures have been compiled as Dataset 1 and are provided as a supplementary file. Uncropped Western blot images are included in the Supplementary Information, along with supplementary methods and supplementary figures.

## Abbreviations

AMPK: AMP-activated protein kinase
Arg1: arginase
ASA: argininosuccinate
ASL: argininosuccinate lyase
ATP: adenosine triphosphate
BBB: blood–brain barrier
CIRI: cerebral ischemia-reperfusion injury
G6P: glucose-6-phosphate
IL-6: interleukin-6
IL-10: interleukin-10
IL-1β: interleukin 1 beta
iNOS: inducible nitric oxide synthase
LPO: lipid hydroperoxide
MCAO: middle cerebral artery occlusion
MDA: malondialdehyde
OGD/R: Oxygen–glucose deprivation and reoxygenation
OXPHOS: oxidative phosphorylation
PPP: pentose phosphate pathway
ROS: reactive oxygen species
SOD: superoxide dismutase
TCA: tricarboxylic acid
TNF-α: tumor necrosis factor alpha
VK: Vespakinin-M
