# Supplementary material for: Vespakinin-M delineates an AMPK/mTOR-arginine-TCA cycle axis to act as an immunometabolic switch in post-stroke microglia
Source: Redox Biol. 2026 May 12;94:104210. doi: 10.1016/j.redox.2026.104210 (PMC13214344; doi:10.1016/j.redox.2026.104210)
Supplement: Multimedia component 1 [file mmc1.pdf]

Supplementary Material

Vespakinin-M delineates an AMPK/mTOR–arginine–TCA cycle axis to act as an immunometabolic switch in post-stroke microglia

Dexiao Wang[a,d#], Jingyu Zhang[a,d#], Zhejun Zhuang[a,d#], Qian Wang[a,d], JieLi[a,d], Xue Wang[a,d], Kunkun Li[a,d], Yunyun Liu[a,d], Yanhui Cao[a,d], Lijuan Li[a,c], Yunwu Zhang[b], Yu Zhao[a,d], Yingjun Zhao[b*], Hairong Zhao[a,b,d*], Chenggui Zhang[a,d*]

[a]Yunnan Provincial Key Laboratory of Entomological Biopharmaceutical R&D, College of Pharmacy, Dali University, Dali, Yunnan, PR China

[b]Department of Neurology and Department of Neuroscience, the First Affiliated Hospital of Xiamen University, Institute of Neuroscience, Fujian Provincial Key Laboratory of Neurodegenerative Disease and Aging Research, School of Medicine, Xiamen University, Xiamen, Fujian, China

[c]The First Affiliated Hospital of Dali University, Yunnan, PR China

[d]National-Local Joint Engineering Research Center of Entomoceutics, Dali, PR China

[#]These authors contributed equally to this work.

[*]Corresponding authors.

E-mail addresses: Yingjun Zhao (yjzhao@xmu.edu.cn); Hairong Zhao (hr_zhaoxmu@126.com), Chenggui Zhang (chenggui_zcg@hotmail.com).

1. Supplementary materials and methods

1.1 The 2,3,5-Triphenyltetrazolium chloride (TTC) staining

At indicated time points after MCAO/R, mice were euthanized and brains were rapidly removed. Brains were sliced into 1-mm coronal sections using a mouse brain matrix. Sections were incubated in 2% TTC solution (Sigma-Aldrich, USA) in PBS at 37°C for 15 min in the dark, then fixed in 4% paraformaldehyde. Infarct area (pale white) was measured using ImageJ software, and infarct volume was calculated as percentage of contralateral hemisphere volume after correction for edema, as described previously.

1.2 Biochemical assays for oxidative stress markers

The ischemic hemisphere was homogenized in ice-cold PBS, and the supernatant was collected after centrifugation. Protein concentration was determined using a BCA assay kit for normalization. Superoxide dismutase (SOD) activity, malondialdehyde (MDA) content, lipid hydroperoxide (LPO) content, and glutathione peroxidase (GSH-Px) activity in brain tissue homogenates were measured using respective colorimetric/fluorometric assay kits (#BC5165, #BC6415, # BC5245, # BC1175, Solarbio, China) according to the manufacturer's instructions. Absorbance or fluorescence was recorded using a microplate reader. Enzyme activities were expressed as units per mg protein (U/mg prot), and metabolite levels as nmol per mg protein (nmol/mg prot).

1.3 Cell viability and cytotoxicity assay (CCK-8)

Primary microglia were seeded in 96-well plates (1×10^3 cells/well). Cells were treated with VK (1, 2, 4, 8, 16, 32, 64, 128 and 256 μg/mL) for 24, 48, or 72 h; the medium was replaced every 24 h. For the OGD/R experiment, cells were pretreated with VK (0.1–16 μg/mL) for 24 h, subjected to OGD for 6 h, and then reoxygenated for 24 h in the presence of VK. Cell viability was assessed using the CCK-8 reagent. After 1 h incubation at 37°C, absorbance was measured at 450 nm using a microplate reader (SpectraMax M2, Molecular Devices). IC_{50} values were calculated by fitting dose–response curves using nonlinear regression (four-parameter logistic model) with GraphPad Prism 10.4.0.

1.4 Detection of iNOS/Arg1 Content

Ischemic hemispheres from MCAO/R mice were collected and analyzed using commercial ELISA kits for iNOS (#CSB-E08326m, CASUBIO, China) and Arg1 (#CSB-EL002005MO, CASUBIO, China), following the manufacturers' instructions. Briefly, 100 mg of tissue from the ischemic hemisphere was rinsed with 1× PBS to

remove blood contamination, then homogenized in 1 mL of 1× PBS. The homogenate was centrifuged, and the supernatant was collected as the tissue lysate. After sequential incubation with the provided working solutions, the optical density (OD) was measured at 450 nm using a microplate reader. Protein concentrations were calculated based on standard curves generated for each assay.

1.5 Detection of L-LDH Content/protein expression

BV2 cells/primary microglia were pretreated with VK for 24 h before establishment of the OGD/R model. Cells were collected into centrifuge tubes, lysed with L-LDH extraction buffer via ultrasonication, and centrifuged to obtain the supernatant as the test sample. L-LDH levels were determined using an L-LDH assay kit (#BC0685, Solarbio, China). The corresponding working solution was added, a standard curve was prepared, and the absorbance was finally measured at 450 nm.Formula: L-LDH (U/10^4 cells) = 66.67 × sample concentration / cell number × dilution factor.

In addition, protein was extracted from the treated primary microglia, and western blot was performed to detect the expression of LDHA (1:1000, #3582, CST, USA).

1.6 Intracellular ROS measurement

Primary microglia were seeded in black-walled 96-well plates (8×10^3 cells/well) and pretreated with VK for 24 h. Following OGD/R (as in Section 2.11), cells were incubated with 10 μM DCFH-DA (#R253, Dojindo) at 37°C for 30 min. After washing, fluorescence was measured at 488/525 nm using a microplate reader. Representative images were acquired using a fluorescence microscope (FITC channel).

1.7 Detection of NO Content

Primary microglia were pretreated with VK for 24 h before the OGD/R model was established. The extraction buffer was added, and the supernatant was collected after ultrasonication and centrifugation for subsequent detection. Mice were subjected to MCAO/R surgery and administered VK (150 μg/kg) for 3 consecutive days. The ischemic brain hemispheres were harvested, homogenized with extraction buffer, and centrifuged to obtain the supernatant. NO concentration was measured using an NO assay kit (#BC1475, Solarbio, China). The working solution and test samples were mixed, and the absorbance was detected at 550 nm.

Formula: NO content (μmol/10^4 cells) / (μmol/g) = 0.05 × (A test – A blank) / (A standard – A blank) / cell number / sample weight.

1.8 Untargeted metabolomics profiling of primary microglia

Primary microglia were divided into four groups: (1) control (normal culture), (2) OGD/R, (3) OGD/R + VK (2 μg/mL), and (4) OGD/R + VK (4 μg/mL). Cells were pretreated with VK for 12 h, then subjected to OGD for 6 h followed by reoxygenation. Approximately 1×10^7 cells per dish were used for metabolomic analysis. After treatment, cells were washed three times with ice-cold PBS. One milliliter of extraction solvent (methanol/acetonitrile/water, 2:2:1, v/v/v) was added directly to each 6-cm dish, and cells were scraped and transferred to 1.5-mL microcentrifuge tubes. The samples were vortexed for 30 s and sonicated on ice for 10 min. Subsequently, the samples underwent three freeze–thaw cycles (freezing in liquid nitrogen followed by ice-bath sonication for 10 min each), then incubated at −20°C for 1 h to precipitate proteins. After centrifugation at 13,000 rpm for 15 min at 4°C, 800 μL of supernatant was collected and dried under vacuum using a centrifugal concentrator. The dried extracts were stored at −80°C until LC-MS/MS analysis. Untargeted metabolomics analysis was performed following the same LC-MS/MS parameters and data processing procedures described in Section 2.26.

1.9 Arginine deprivation and supplementation assay

Primary microglia were seeded in culture plates. After 24 h, cells were divided into three groups: the Control group (maintained in normal medium), the Arg^- group (cultured in arginine-deficient medium, #MA0545, GMeilunBio), and the VK group (cultured in arginine-deficient medium supplemented with 4 μg/mL VK). Following 24 h of treatment, the medium in the Arg^- and VK groups was replaced with normal medium to reintroduce arginine. After 6 h of recovery, cells were harvested for downstream analyses, including measurement of intracellular arginine, ATP, NAD^+/NADH ratio, Arg1 protein expression, and gene expression of arginine metabolism-related markers.

1.10 Bio-layer interferometry affinity assay

Recombinant His-tagged AMPK protein was immobilized on a Ni-NTA sensor chip. After equilibration and baseline correction in PBS buffer (pH 7.4, containing 0.01% Tween-20 and 0.1% BSA), the chip was immersed in VK solutions (3.13, 6.25, 12.50, 25.00, 50.00, 100.00, 200.00 μM) of gradient concentrations for association-dissociation kinetic detection (25°C, 3 biological replicates). The 1:1 Langmuir binding model was fitted using Octet Data Analysis Software 12.0, and the dissociation constant (K) was calculated after subtracting the background signal.

1.11 Colocalization analysis of immunofluorescence images

Immunofluorescence images were acquired using a confocal microscope (Olympus VS200) with identical exposure settings across all samples. Colocalization analysis was

performed using ImageJ software (NIH, USA) as follows. First, raw multi-channel images were background-subtracted and the scale was calibrated. The two channels of interest (Glut1 with IBA1 or NeuN; PFKFB3 with IBA1 or NeuN) were split and then merged. For each region of interest (ROI), a straight line was drawn across the target structure (e.g., microglia-neuron interface) using the line tool. The 'Plot Profile' function was then applied to generate fluorescence intensity distribution curves for both markers along the ROI path. The x-axis represents the distance along the ROI (in μm), and the y-axis represents the fluorescence intensity (arbitrary units). The resulting intensity profiles were visualized and analyzed using GraphPad Prism 10.4.0. At least three random fields per group were analyzed, and representative curves were shown.

2. Supplementary Figure

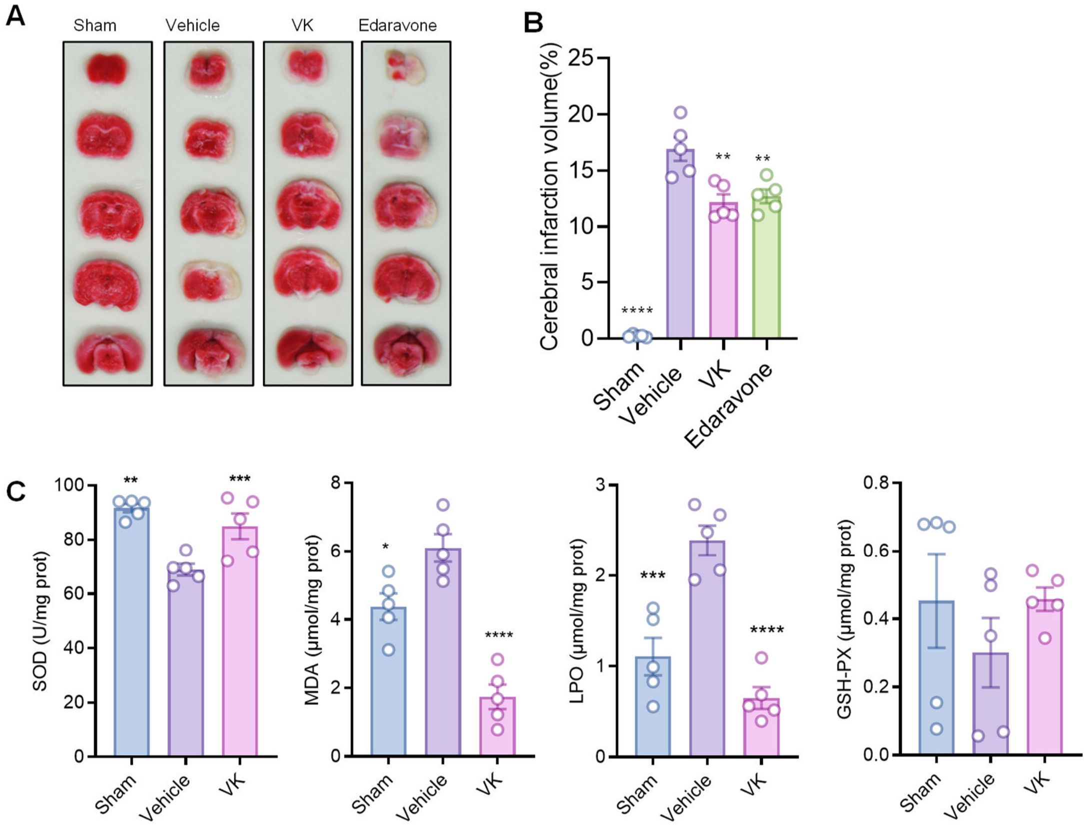

Supplementary Fig. 1. Vespakinin-M (VK) reduces cerebral infarct volume following cerebral ischemia–reperfusion injury (CIRI). (A) Representative images of 2,3,5-triphenyltetrazolium chloride (TTC) staining of coronal brain sections from mice subjected to sham operation, middle cerebral artery occlusion/reperfusion (MCAO/R) + vehicle, or MCAO/R + VK treatment (150 μg/kg, i.p.). Viable tissue stains red, while infarcted areas appear pale; (B) Quantification of infarct volume expressed as percentage of total hemispheric volume; (C) Superoxide dismutase (SOD) activity, malondialdehyde (MDA) content, lipid hydroperoxide (LPO) content, and glutathione peroxidase (GSH-Px) activity in brain tissue. Data are presented as mean ± SEM; n=6 biologically independent mice per group. Statistical significance was determined by one-way ANOVA followed by Bonferroni's post hoc test: $^{*}P<0.05$, $^{**}P<0.01$, $^{***}P<0.001$, $^{****}P<0.0001$ versus the MCAO/R+Vehicle group.

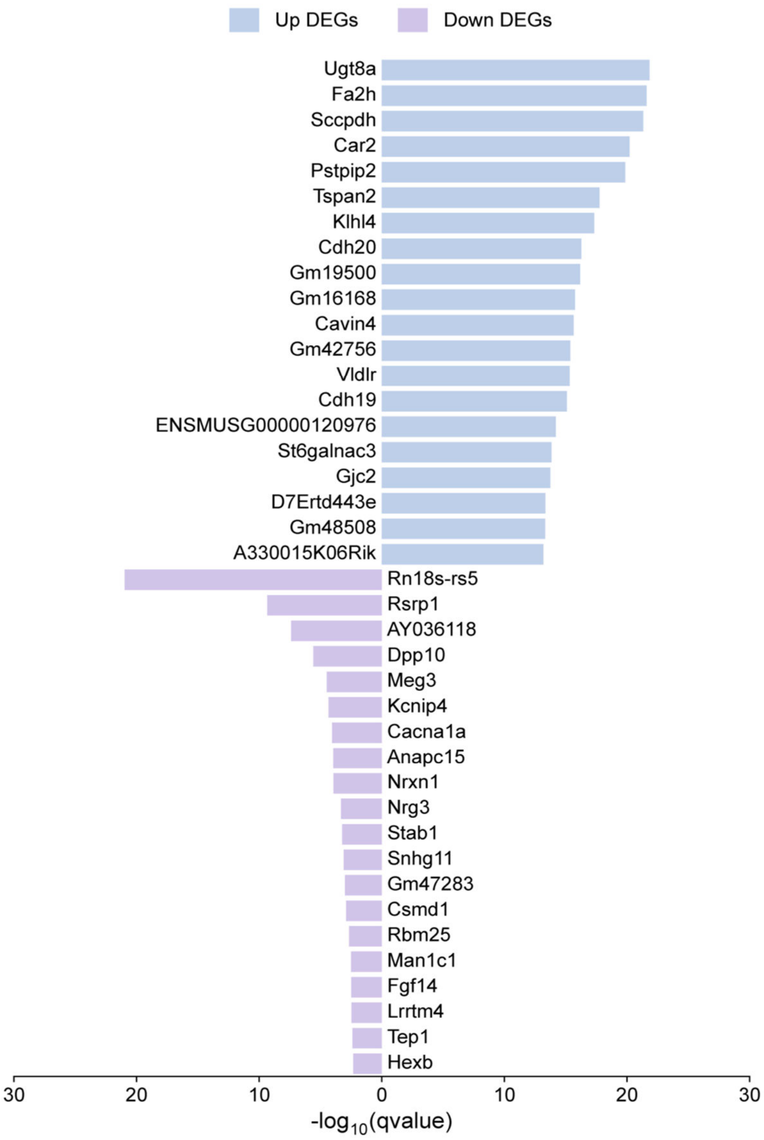

Supplementary Fig. 2. Bidirectional bar plot of the top 20 differentially expressed genes (DEGs) in Fig. 1H. This figure displays the top 20 significantly altered genes in microglia from VK-treated versus MCAO/R vehicle group mice, identified by single-cell RNA sequencing (scRNA-seq) analysis of the ischemic hemisphere. DEGs were filtered using the standardized criteria: $|\log_2$ (Fold Change) $|>1$ and adjusted $P<0.05$ (Benjamini-Hochberg false discovery rate correction).

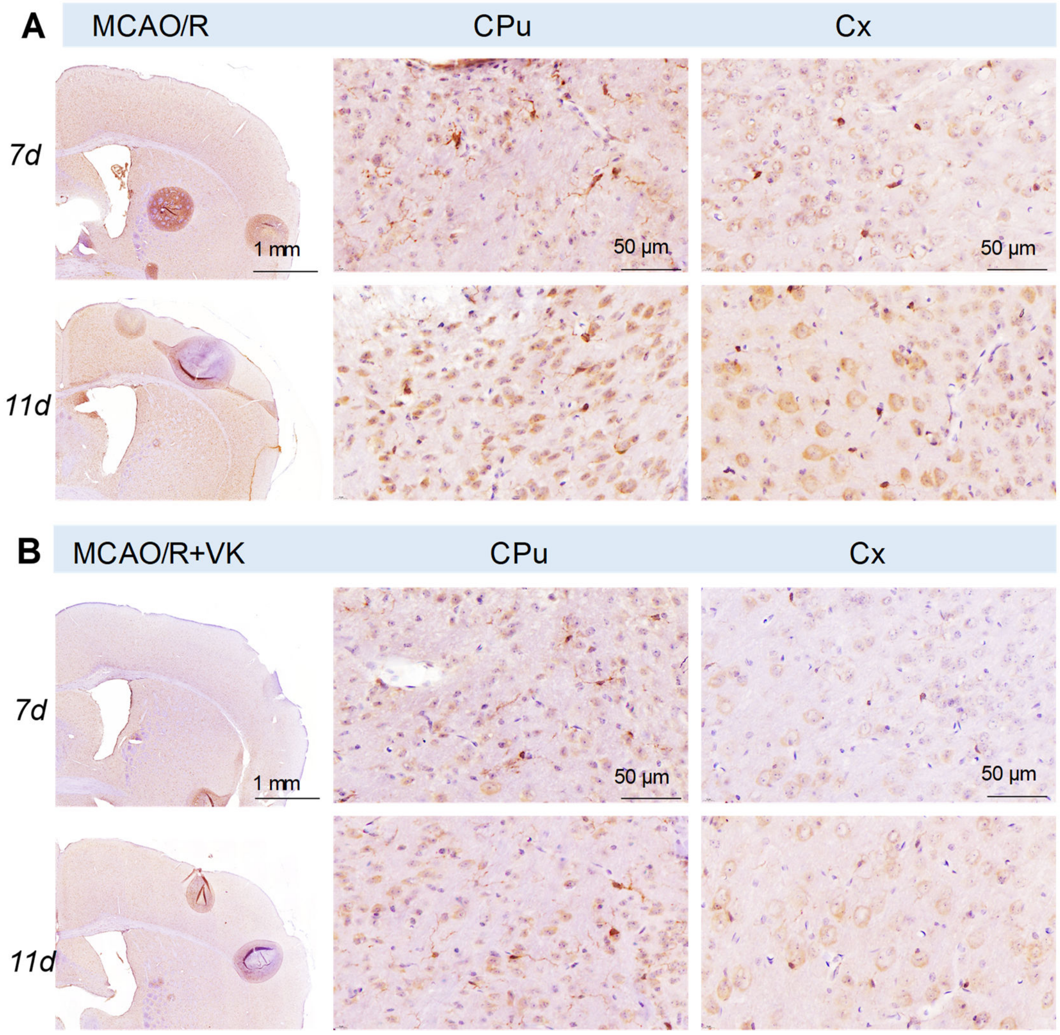

Supplementary Fig. 3. Immunohistochemical analysis of $IBA1^{+}$ microglia at later time points after MCAO/R.Representative images showing Iba1-positive microglia in the caudate putamen (CPu) and cerebral cortex (Cx) of (A) MCAO/R vehicle-treated and (B) VK-treated mice at 7 and 11 days post-MCAO/R. Scale bars: 1 mm (overview), 50 μm (inset).

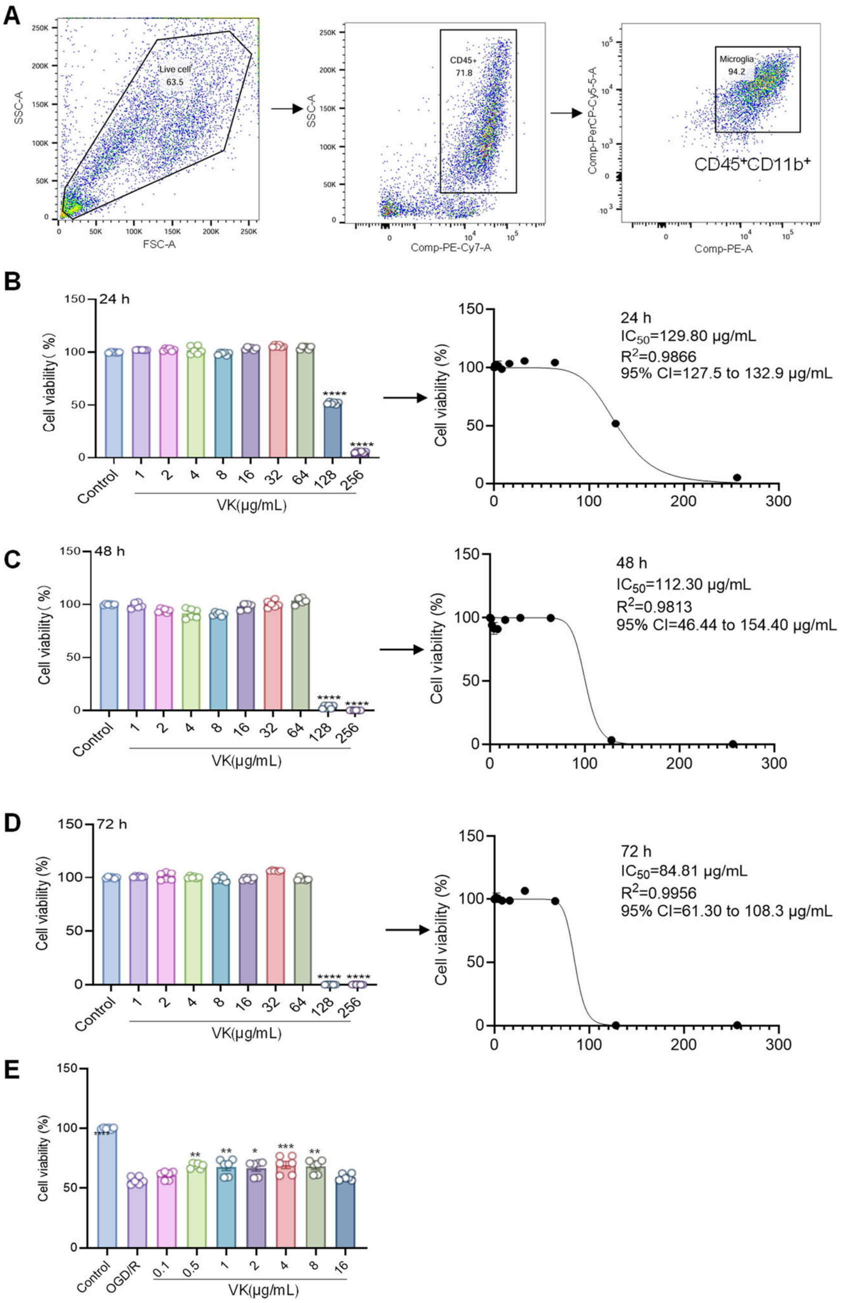

Supplementary Fig. 4. Characterization of primary microglia and determination of VK's safe/effective concentrations. (A) Identification of primary microglia by flow cytometry using surface markers CD11b and CD45. A representative plot (from three independent isolations) shows the gated CD11b$^+$CD45$^+$ population. (B–D) Cytotoxicity of VK (1–256 μg/mL) on primary microglia after 24, 48, and 72 h exposure, assessed by CCK-8 assay. (E) VK pretreatment (0.5–16 μg/mL) protects microglia against OGD/R-induced loss of viability. Data information (B–E): Values are mean ± SEM (n=6 biological replicates). $^{**}P < 0.0001$ vs. untreated control (B–D); $^{**}P < 0.05$, $^{***}P < 0.01$, $^{***}P < 0.001$, $^{****}P < 0.0001$ vs. OGD/R (0 μg/mL VK) group (E). Statistics: two-way ANOVA with Bonferroni's post-hoc test.

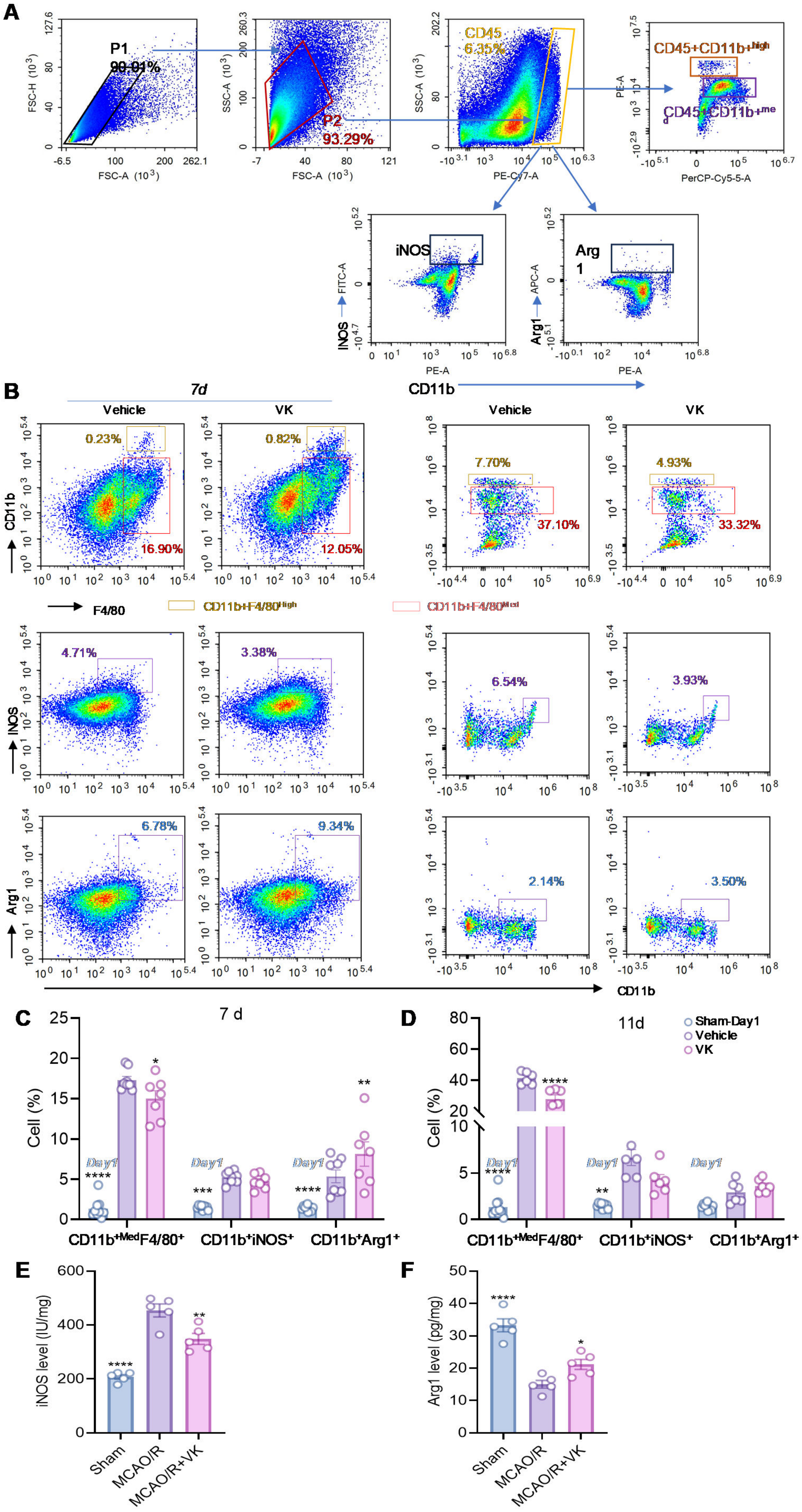
A
P1
90.91%
P2
93.29%
CD45
6.35%
CD45+CD11b+high
FSC-A (10³)
SSC-A (10³)
PE-Cy7-A
PerCP-Cy5-5-A
iNOS
Arg 1
CD11b
B
7d
Vehicle
VK
F4/80
CD11b+F4/80High
0.23%
16.90%
0.82%
12.05%
7.70%
37.10%
4.93%
33.32%
4.71%
3.38%
6.54%
3.93%
6.78%
9.34%
2.14%
3.50%
C
7 d
Cell (%)
D
11d
Sham-Day1
Vehicle
VK
E
iNOS level (IU/mg)
Sham
MCAO/R
MCAO/R+VK
F
Arg1 level (pg/mg)

Supplementary Fig. 5. Flow cytometric analysis of microglial M1/M2 phenotypes at 7 and 11 days post-MCAO/R and ELISA validation of iNOS/Arg1 expression. (A) Gating strategy for flow cytometric identification of microglial subsets: live cells → singlets →$CD45^+$→ $CD45^+CD11b^+$→ $iNOS^+$ (M1) and $Arg1^+$ (M2). (B–D) Representative flow cytometry plots showing M1 ($iNOS^+$) and M2 ($Arg1^+$) microglial subsets at 7 and 11 days post-MCAO/R. Note: Sham group was measured only on day 1; its values on days7 and 11 are the day 1 data repeated for visual reference (not independent sampling). (E, F) Quantification of iNOS (E) and Arg1 (F) protein levels in the ischemic hemisphere by ELISA. Statistical significance was determined by two-way ANOVA followed by Bonferroni's post hoc test: $^{*}P<0.05$, $^{**}P<0.01$, $^{***}P<0.001$, $^{****}P<0.0001$ versus the MCAO/R group at the corresponding time point.

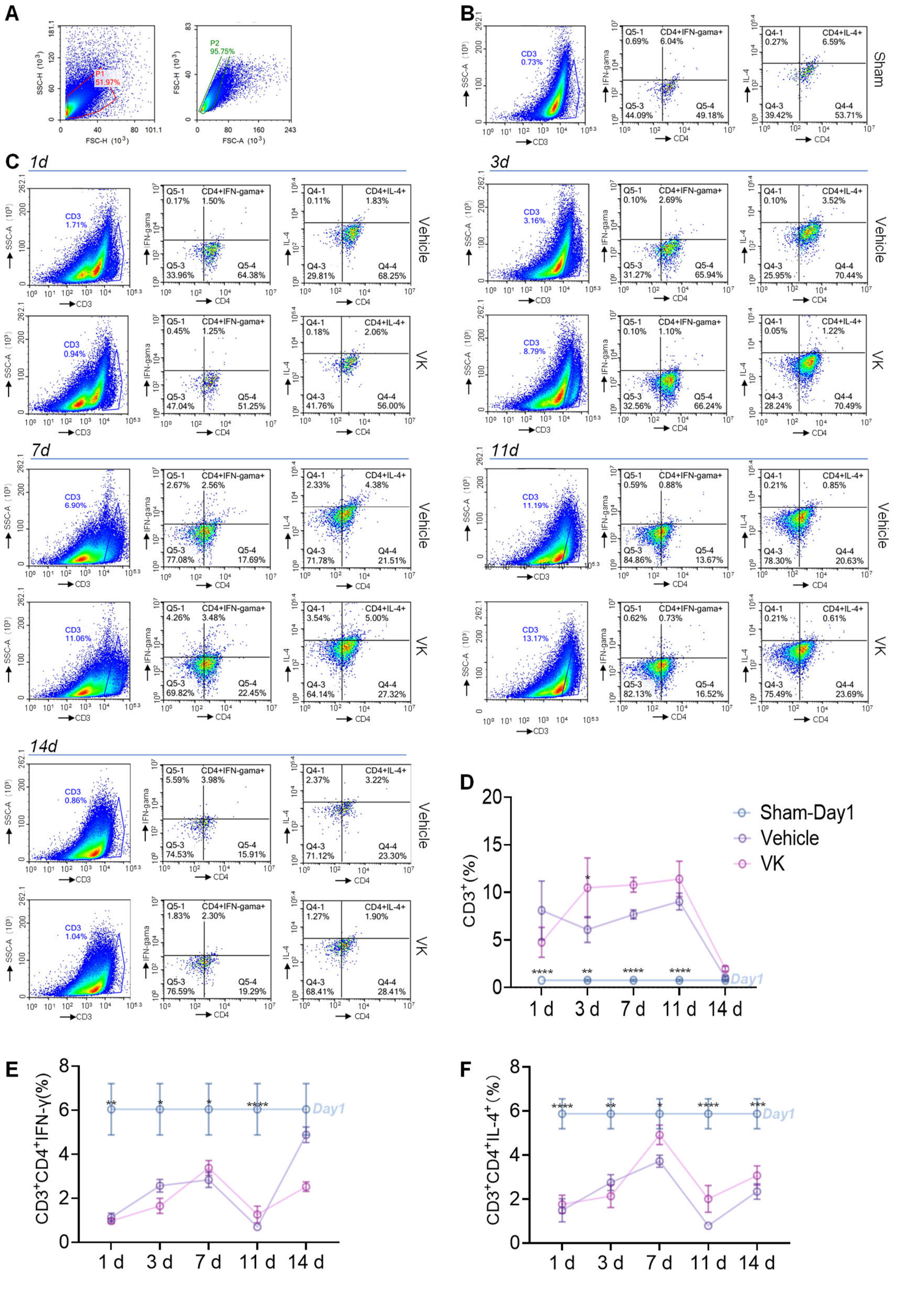

Supplementary Fig. 6. Flow cytometric analysis of microglia-associated T helper cell subsets following MCAO/R. (A) Gating strategy for identifying microglia and associated T cells in the ischemic hemisphere. Live single cells were sequentially gated for CD45⁺CD11b⁺ (microglia/myeloid cells) and CD3⁺ (T cells) populations; (B, C)

Representative flow cytometry plots showing the distribution of $CD4^+$ T cells and their intracellular cytokine expression (IFN-γ and IL-4) at different time points post-MCAO/R in the indicated treatment groups; (D) Quantification of total microglia-associated T cells ($CD45^+CD11b^+CD3^+$); (E) Quantification of T helper 1 (Th1)-polarized cells, defined as $CD45^+CD11b^+CD3^+CD4^+IFN\text{-}\gamma^+$; (F) Quantification of T helper 2 (Th2)-polarized cells, defined as $CD45^+CD11b^+CD3^+CD4^+IL\text{-}4^+$. *Note: Sham group was measured only on day 1; its values on days3, 7, 11, and 14 are the day 1 data repeated for visual reference (no independent sampling).* Data are presented as mean ± SEM; n=6 biologically independent mice per group. Statistical significance was determined by two-way ANOVA followed by Bonferroni's post hoc test: $^{*}P<0.05$, $^{**}P<0.01$, $^{***}P<0.001$, $^{****}P<0.0001$ versus the vehicle group at the corresponding time point.

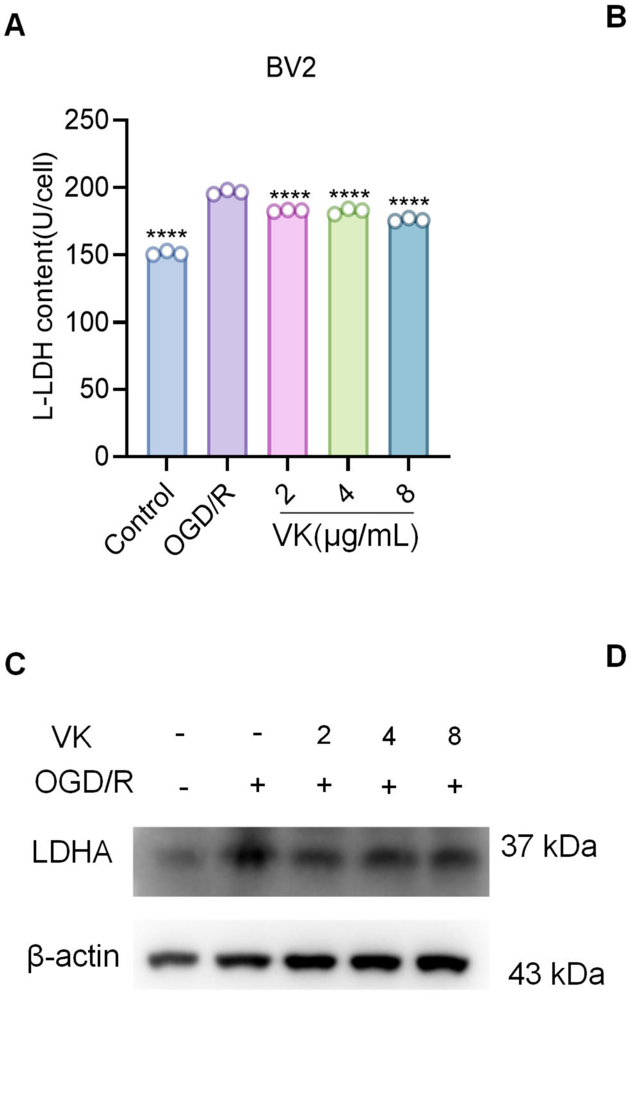

Supplementary Fig. 7. Detection of intracellular L-lactate dehydrogenase (L-LDH) content in microglia. (A) L-LDH content in BV2 cells measured by a commercial colorimetric assay. (B) L-LDH content in primary microglia measured by the same assay. (C) LDHA protein expression detected by Western blot. (D) Densitometric quantification of (C), normalized to loading control. Data are mean ± SEM from three independent experiments. One-way ANOVA with Bonferroni's post hoc test versus OGD/R group: $^{*}P<0.05$, $^{**}P<0.01$, $^{***}P<0.001$, $^{****}P<0.0001$ versus the OGD/R group.

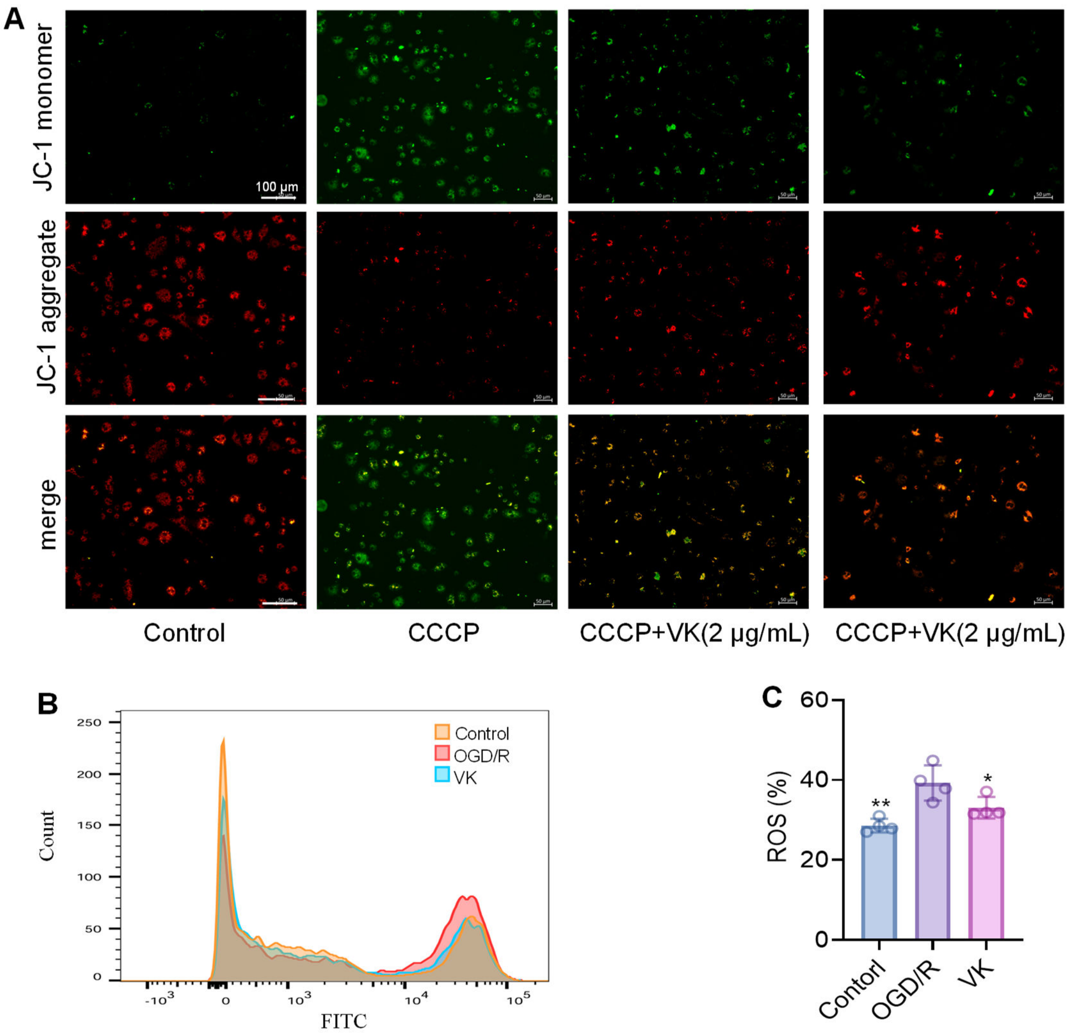

Supplementary Fig. 8 VK alleviates oxidative stress and preserves mitochondrial membrane potential in OGD/R-challenged microglia. (A) Mitochondrial membrane potential (ΔΨm) measured by JC-1 staining, where the red/green fluorescence ratio indicates the proportion of polarized to depolarized mitochondria. (B) Representative flow cytometry plots of intracellular ROS levels detected by DCFH-DA. The histogram defines the ROS-positive gate. (C) Quantification of ROS-positive cells from (B). Data are presented as mean ± SEM from n=3 independent biological experiments (primary microglial isolations), each with technical replicates. Statistical significance was determined by one-way ANOVA followed by Bonferoni's post hoc test: $^{*}P<0.05$, $^{**}P<0.01$, $^{***}P<0.001$, $^{****}P<0.0001$ versus the OGD/R group.

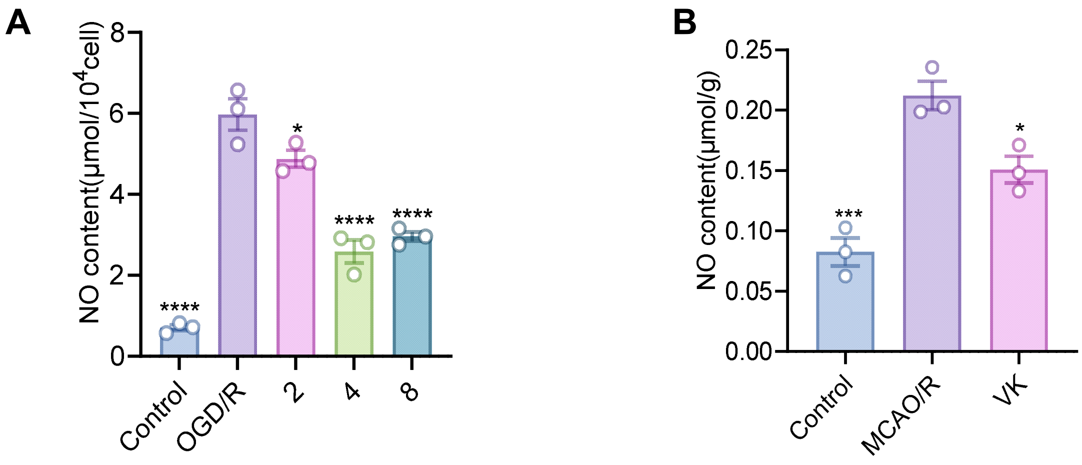

Supplementary Fig. 9 VK markedly inhibits the abnormal increase of NO after CIRI. (A) Intracellular NO content in primary microglia;(B) NO content in mouse brain tissue. Data are presented as mean ± SEM from n=3 independent biological experiments (primary microglial isolations), each with technical replicates. Statistical significance was determined by one-way ANOVA followed by Bonferroni's post hoc test: $^{*}P<0.05$, $^{**}P<0.01$, $^{***}P<0.001$, $^{****}P<0.0001$ versus the OGD/R (0 μg/mL VK) group.

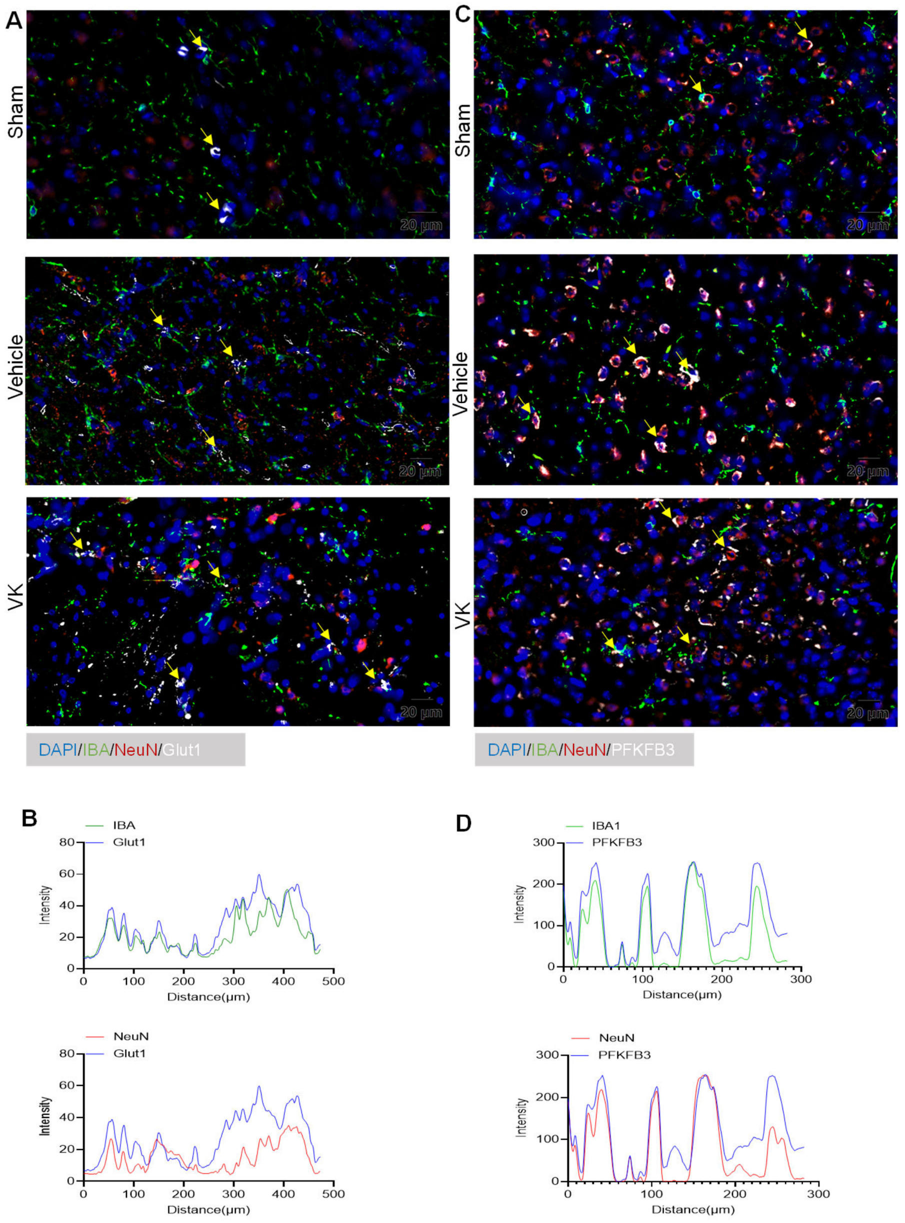

Supplementary Fig. 10 VK restores glucose metabolism markers at the microglia-neuron interface after MCAO/R. Representative immunofluorescence images of the peri-infarct cortex at 7 days post-MCAO/R, showing co-staining for microglia (IBA1, green), neurons (NeuN, red), and (A) the glucose transporter Glut1 (magenta) or (C) the glycolytic enzyme PFKFB3 (magenta); (B) and (D) show the co-localization coefficients of Glut1/PFKFB3 with microglia and NeuN in (A) and (C), respectively. Nuclei are counterstained with DAPI (blue). Insets show magnified views of the boxed regions, with arrowheads indicating representative cells exhibiting co-localization at the microglia-neuron interface. Scale bars: 20 μm (overview).

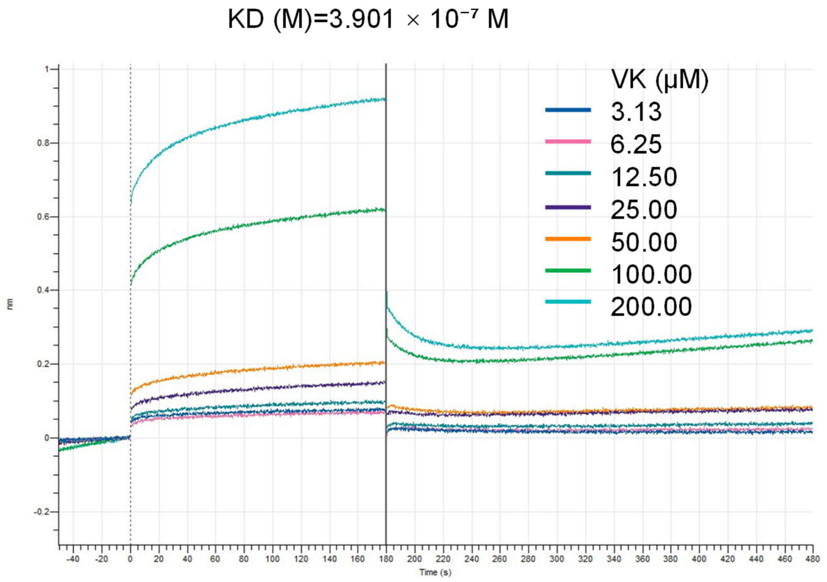

Supplementary Fig. 11 Bio-layer interferometry (BLI) demonstrates direct binding between Vespakinin-M (VK) and AMPK.

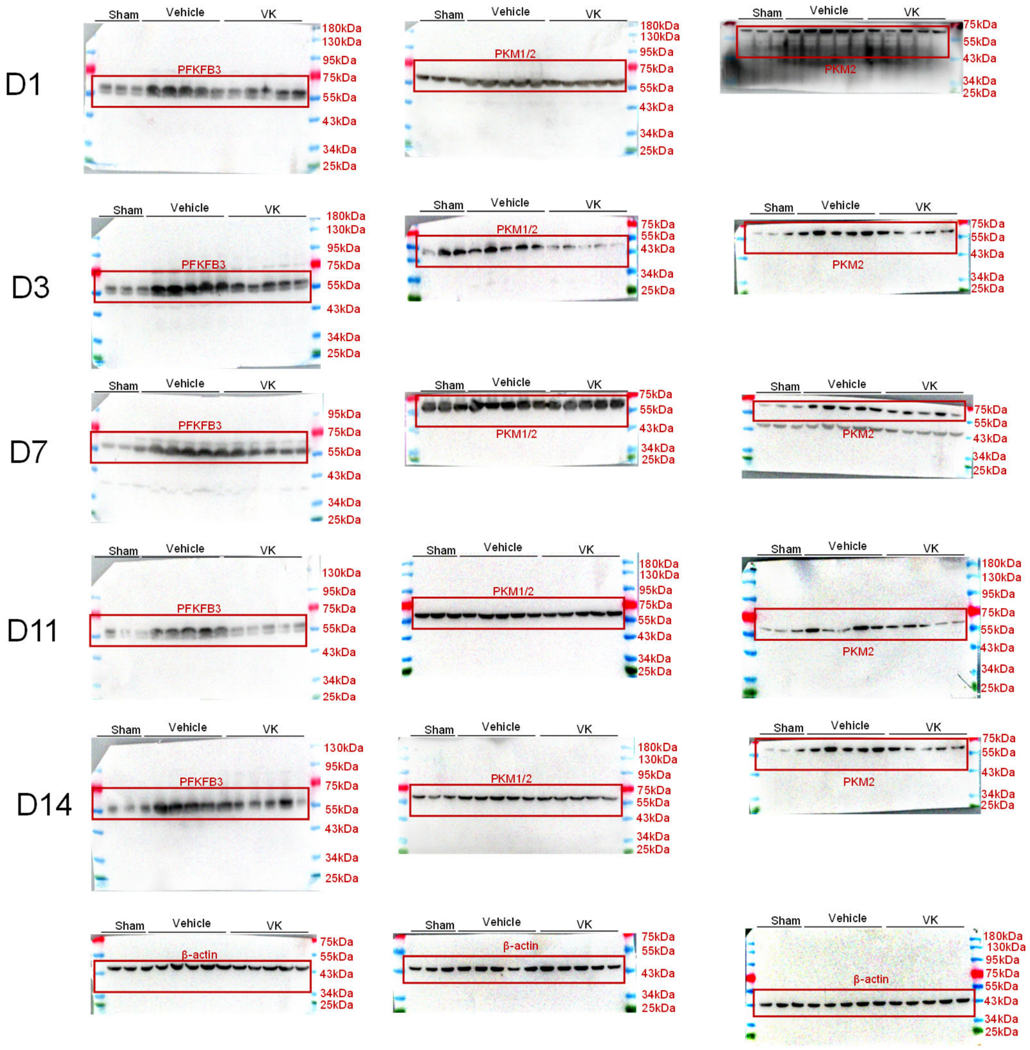

Supplementary Fig. 12 Uncropped blots referring to Fig. 6A–C

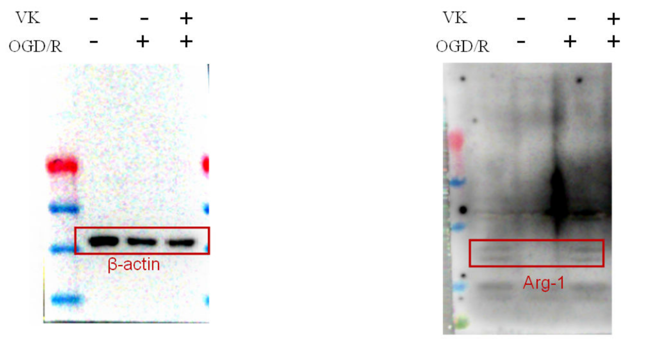

Uncropped blots referring to Fig.7B

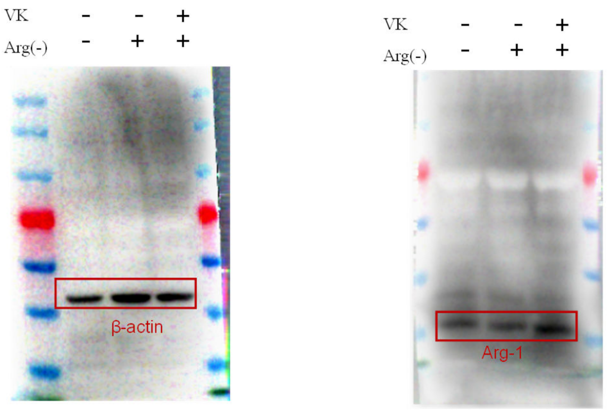

Uncropped blots referring to Fig.7E

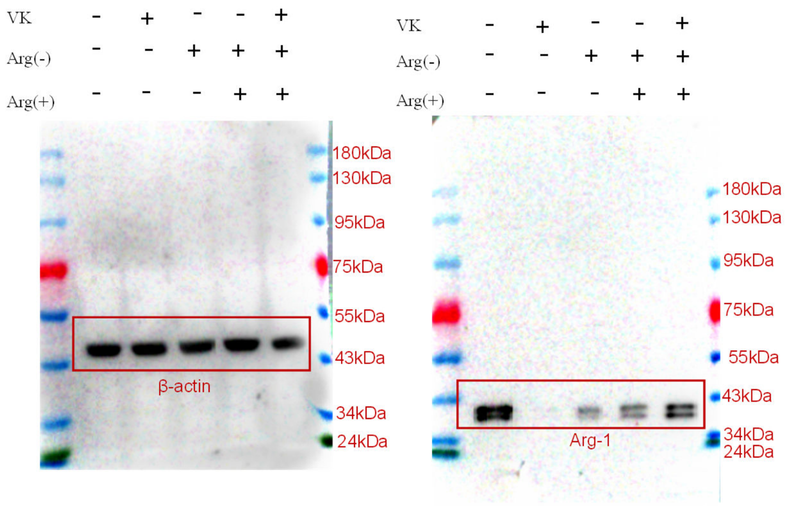

Uncropped blots referring to Fig.7L

Supplementary Fig. 13 Uncropped blots referring to Fig. 7

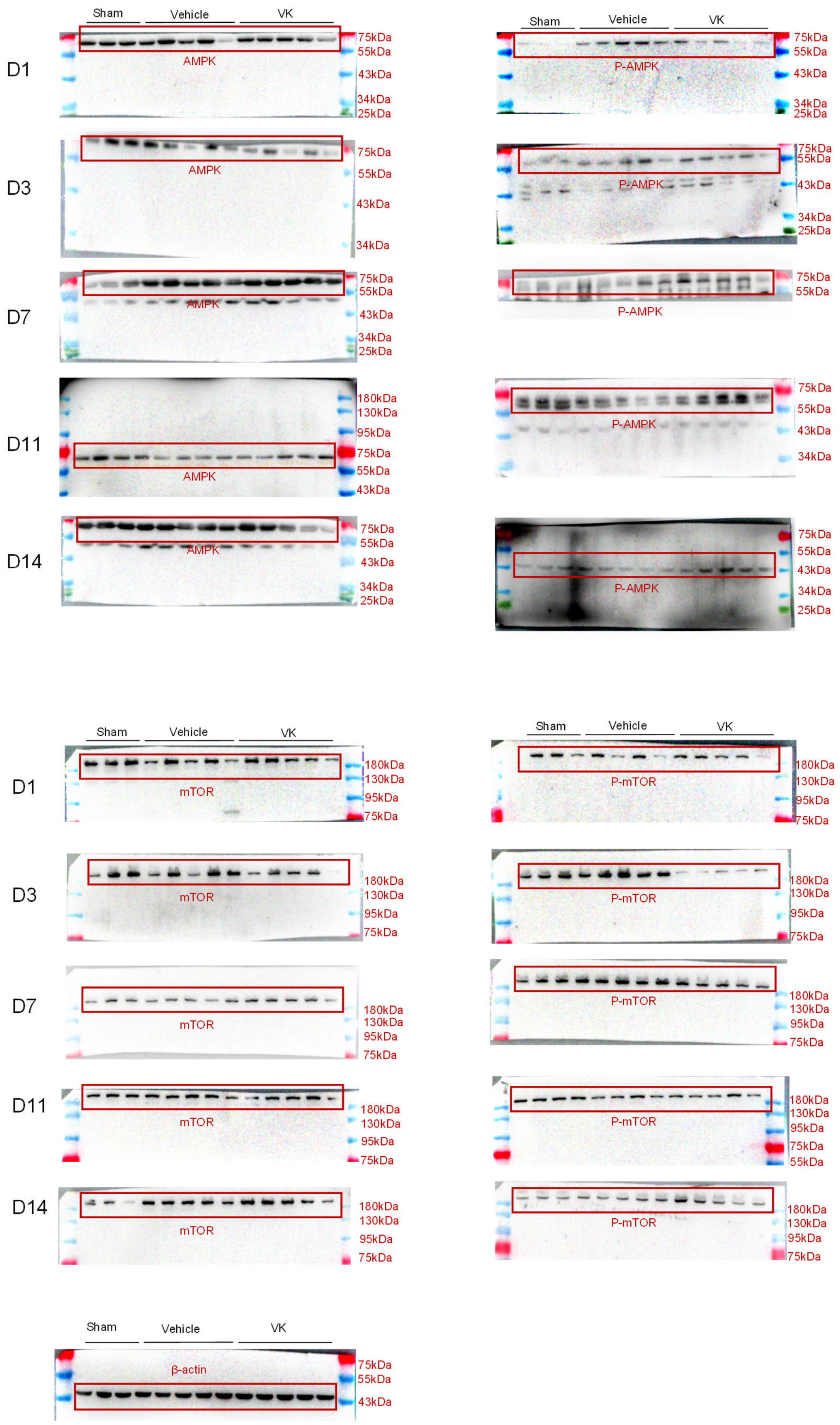

Supplementary Fig. 14 Uncropped blots referring to Fig. 9A and B

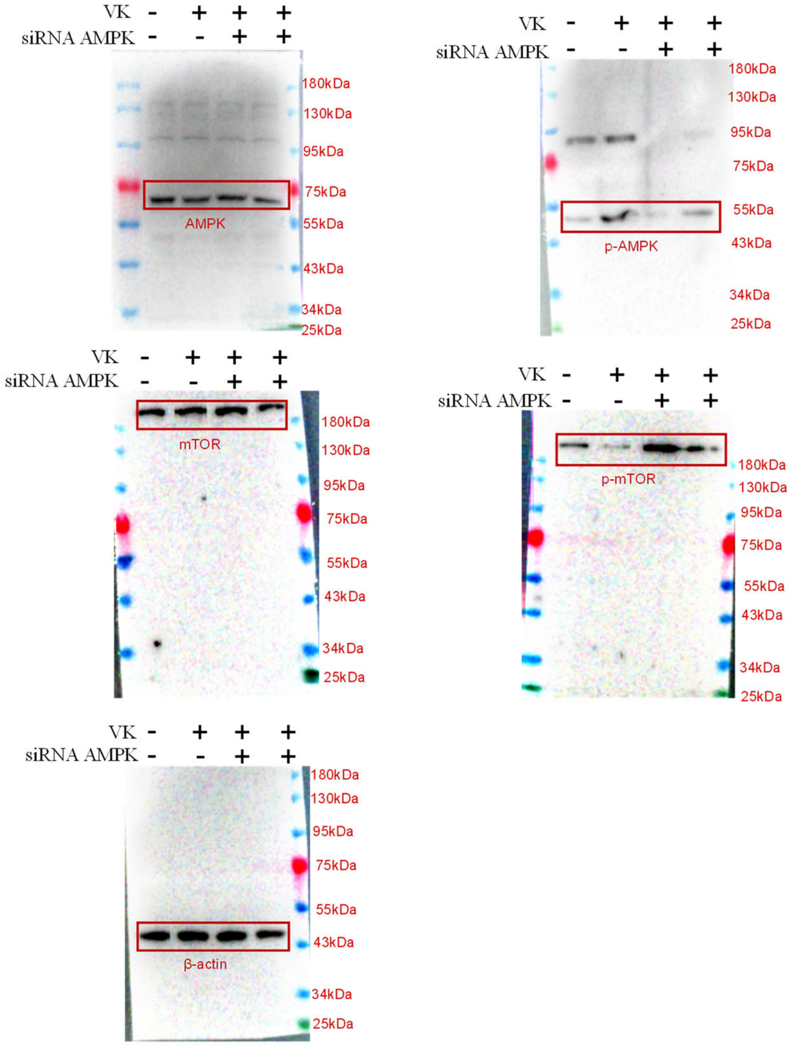

Uncropped blots referring to Fig.9E

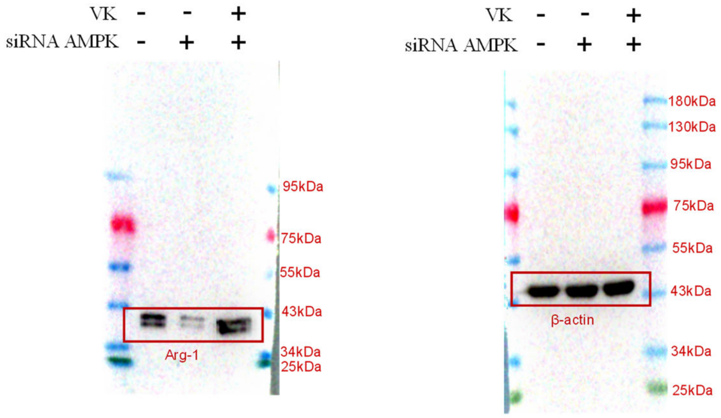

Uncropped blots referring to Fig.9H

Supplementary Fig. 15 Uncropped blots referring to Fig. 9E and H

3. Supplementary Tables

Supplementary Table 1 Detailed list of mice used in this study.

Experiment Name	Mouse Strain	Age	Number
Primary microglial cell cultures	C57BL/6	1−3 days	50
Immunohistochemical staining	C57BL/6	8−10 weeks	65
Immunoblotting	C57BL/6	8−10 weeks	65
Flow cytometric analysis of microglial polarization	C57BL/6	8−10 weeks	85
ELISA—Inflammatory Cytokine Detection	C57BL/6	8−10 weeks	40
ELISA—Oxidative Stress Marker Detection	C57BL/6	8−10 weeks	20
TEM analysis of mitochondrial ultrastructure	C57BL/6	8−10 weeks	10
Untargeted Metabolomics	C57BL/6	8−10 weeks	20
Proteomic	C57BL/6	8−10 weeks	16
Single-cell RNA Sequencing	C57BL/6	8−10 weeks	6
ELISA—Arg1/ iNOS Detection	C57BL/6	8−10 weeks	18
Total			395
